# Aptamer-Based Strategies for Colorectal Cancer Detection: Emerging Technologies and Future Directions

**DOI:** 10.3390/bios15110726

**Published:** 2025-11-01

**Authors:** María Jesús Lobo-Castañón, Ana Díaz-Fernández

**Affiliations:** 1 Departamento de Química Física y Analítica, Universidad de Oviedo, Av. Julián Clavería 8, 33006 Oviedo, Spain; 2 Instituto de Investigación Sanitaria del Principado de Asturias, Avenida de Roma, 33011 Oviedo, Spain; anadf@uniovi.es

**Keywords:** aptamer, CRC biomarkers, liquid biopsy, SELEX

## Abstract

Colorectal cancer (CRC) remains a leading cause of cancer-related morbidity and mortality worldwide, with patient outcomes highly dependent on early and accurate diagnosis. However, existing diagnostic methods, such as colonoscopy, fecal occult blood testing, and imaging, are often invasive, costly, or lack sufficient sensitivity and specificity, particularly in early-stage disease. In this context, aptamers, which are synthetic single-stranded oligonucleotides capable of binding to specific targets with high affinity, have emerged as a powerful alternative to antibodies for biosensing applications. This review provides a comprehensive overview of aptamer-based strategies for CRC detection, spanning from biomarker discovery to clinical translation. We first examine established and emerging CRC biomarkers, including those approved by regulatory agencies, described in patents, and shared across multiple cancer types. We then discuss recent advances in aptamer selection and design, with a focus on SELEX variants and in silico optimization approaches tailored to CRC-relevant targets. The integration of aptamers into cutting-edge sensing platforms, such as electrochemical, optical, and nanomaterial-enhanced aptasensors, is highlighted, with emphasis on recent innovations that enhance sensitivity, portability, and multiplexing capabilities. Furthermore, we explore the convergence of aptasensing with microfluidics, and wearable technologies to enable intelligent, miniaturized diagnostic systems. Finally, we consider the clinical and regulatory pathways for point-of-care implementation, as well as current challenges and opportunities for advancing the field. By outlining the technological and translational trajectory of aptamer-based CRC diagnostics, this review aims to provide a roadmap for future research and interdisciplinary collaboration in precision oncology.

## 1. Introduction

Colorectal cancer (CRC) is the third most commonly diagnosed malignancy worldwide and the second leading cause of cancer-related mortality. Its incidence is steadily increasing, driven not only by aging populations but also by modified lifestyle factors such as diet, obesity, and physical inactivity, which underscores the need for effective strategies for prevention, early detection, and treatment [1]. Despite advances in therapeutic approaches, prognosis remains strongly stage-dependent, making timely and accurate diagnosis the cornerstone of improved patient outcomes [2].

Population-based screening programs have been implemented in many countries, generally targeting individuals over 45 years of age. These initiatives have successfully reduced mortality where participation is high, yet adherence rates remain highly variable across countries and socioeconomic groups [2]. Current screening relies predominantly on stool-based assays. The fecal immunochemical test (FIT), which detects hidden blood, is widely used due to its non-invasiveness and affordability. More recently, DNA/RNA-based assays have been developed to detect tumor-associated genetic alterations shed into stool samples [3]. U.S. Food and Drug Administration (FDA)-approved test such as *Cologuard*, which detects multiple DNA markers [4], and *ColoSense*, which targets RNA signatures, exemplify this progress. Nevertheless, colonoscopy remains the gold standard for CRC diagnosis, as it allows direct visualization of the colon, detection of early lesions, and removal of precancerous polyps [5].

Despite these advances, the sensitivity and specificity of current non-invasive tests remain imperfect. FIT achieves approximately 70–75% sensitivity and 90–95% specificity, while multitarget stool DNA assays such as *Cologuard* reach ~92% sensitivity but lower specificity (~87%) [3,4,5]. This performance, although clinically valuable, leaves room for improvement, particularly for early-stage CRC and precancerous lesions. These limitations have spurred increasing interest in alternative blood-based diagnostic strategies that could offer comparable or superior accuracy with higher patient compliance.

Current CRC screening approaches, including stool-based tests and colonoscopy, still face major challenges that limit their effectiveness in real-world settings. Participation in stool-based screening remains suboptimal, often hindered by lack of awareness, socioeconomic barriers, or perceived inconvenience [6]. FIT lacks sensitivity for advanced adenomas and early-stage cancers, while molecular stool tests, although more sensitive, are costly and not universally accessible. Colonoscopy, despite its comprehensiveness, is invasive, expensive, and associated with low patient compliance [7]. These limitations highlight a persistent gap between technical capability and public health impact. In this context, liquid biopsy approaches, based on the detection of circulating tumor-derived material in biological fluids (e.g., blood, saliva, urine) are emerging as promising alternatives that may enhance compliance and broaden screening coverage [8]. By enabling minimally invasive detection of circulating tumor DNA, RNA, proteins, or extracellular vesicles, liquid biopsy holds potential to overcome many of the challenges faced by conventional screening modalities.

Within this landscape, aptamers have gained considerable attention as versatile recognition elements that could accelerate the development of next-generation liquid biopsy strategies [9]. These short, single-stranded nucleic acids fold into defined three-dimensional structures, enabling them to bind a wide spectrum of molecular targets with high affinity and specificity. Compared with antibodies, aptamers offer several practical advantages: they can be generated entirely in vitro through iterative selection (SELEX), are chemically stable under a broad range of conditions, and can be synthesized at scale with high reproducibility and relatively low cost [10]. Their modular nature also facilitates conjugation with nanomaterials, fluorophores, or electroactive compounds, expanding their functionality in biosensing applications.

Importantly, the adaptability of aptamers makes them well suited for integration into liquid biopsy platforms. They can be tailored to capture circulating tumor biomarkers, thus directly addressing the biomarker diversity highlighted in current CRC research. Combined with advances in nanotechnology, microfluidics, and biosensor engineering, aptamer-based systems are emerging as promising tools for highly sensitive, rapid, and minimally invasive CRC diagnostics. While challenges related to clinical validation, standardization, and regulatory approval remain [10], the unique features of aptamers position them as strong candidates to bridge the gap between laboratory innovation and point-of-care implementation.

Previously, comprehensive and high-quality reviews have focused on the use of aptamers for therapeutic applications and their selection through the cell-SELEX strategy [11,12,13]. However, these works predate several important developments in the field. Since 2019, significant progress has been made in expanding the classes of colorectal cancer (CRC) biomarkers recognized by aptamers, including circulating tumor cells, exosomal and stool-derived nucleic acids, and inflammation-associated proteins, as well as in the engineering of miniaturized, multiplexed, and wearable aptasensors. Furthermore, recent studies have introduced new SELEX variants integrating magnetic, microfluidic, and in silico optimization strategies, alongside improved clinical benchmarking for diagnostic validation.

In contrast to earlier reviews, this work provides a comprehensive and up-to-date overview of aptamer-based strategies for CRC detection, emphasizing advances in biosensing platforms, materials engineering, and translational readiness. We first examine CRC-relevant biomarkers, particularly protein targets that serve as foundations for aptamer selection. We then highlight recent advances in SELEX methodologies, aptamer optimization, and their integration into electrochemical and other biosensing platforms. Finally, we discuss the challenges and opportunities for clinical translation and commercialization, emphasizing the potential of aptamer-based diagnostics to complement or even replace current screening tools.

## 2. Colorectal Cancer Biomarkers

**Figure 1:**
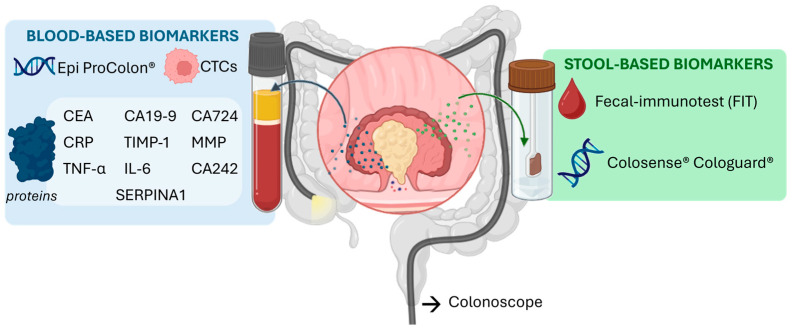
Different types of biomarkers for CRC in stool and blood.

Molecular markers detectable in biological fluids are central to improving the sensitivity and specificity of early cancer detection. They constitute the foundation of liquid biopsy–based strategies, which enable minimally invasive diagnostic testing. In colorectal cancer (CRC), a wide spectrum of circulating biomarkers has been reported [14], including genetic and epigenetic alterations, nucleic acids (DNA and RNA) proteins and circulating tumor cells (Figure 1). In this review, we highlight both clinically validated and emerging CRC biomarkers, as the latter represent promising targets whose translation into practice strongly depends on the development of reliable analytical tools, such as aptamer-based assays. While each class offers valuable diagnostic and prognostic information, proteins remain the most widely implemented in clinical practice and are particularly attractive as targets for aptamer-based recognition due to their abundance, structural diversity, and direct association with tumor biology. The following section provides an overview of the principal biomarker categories, with special emphasis on protein markers as the cornerstone for emerging aptamer-driven diagnostic platforms.

### 2.1. Genetic and Epigenetic Biomarkers

Genetic alterations play a central role in CRC pathogenesis and progression, with recurrent mutations in *APC*, *KRAS*, *TP53*, and *BRAF* among the most extensively studied [15]. *APC* mutations are detected in up to 90% of CRC cases and serve as early events in tumorigenesis [16], whereas *KRAS* and *TP53* alterations are more frequently associated with prognosis and therapy response [17]. Additional recurrent mutations involve *PIK3CA*, *SMAD4*, *NRAS*, and others, though their utility for population screening remains limited [18,19]. Recently, transcriptomic panels such as *ColonSentry*, based on expression profiling of six genes, have been approved for clinical use in high-risk patients to support adherence to colonoscopy with a sensitivity of 78% and a specificity of 66% [20].

Epigenetic changes, particularly DNA methylation, also provide valuable biomarkers [21]. The most established is SEPT9 [22], approved for clinical testing (Epi proColon^®^), which demonstrates high specificity (86–99%) but modest sensitivity (56–79%) for early detection [23]. Other methylation targets, such as NDRG4 and BMP3, are under investigation and incorporated into multi-marker assays.

In addition, microsatellite instability (MSI), arising from defects in mismatch repair genes, represents both a diagnostic marker and a predictor of response to immunotherapy. MSI-high CRCs account for ~15% of cases, particularly in hereditary syndromes, and are increasingly guiding treatment decisions [24].

Taken together, genetic and epigenetic biomarkers provide valuable insights into CRC biology, prognosis, and therapy stratification, but their sensitivity and specificity remain insufficient for widespread use in early, non-invasive screening.

### 2.2. Circulating Nucleic Acids

Circulating tumor DNA (ctDNA), derived from apoptotic or necrotic tumor cells, is increasingly recognized as a promising biomarker in CRC [25]. ctDNA levels correlate with tumor burden, prognosis, and recurrence risk, and are particularly valuable for the detection of molecular residual disease (MRD) after surgery, enabling more precise, stage-independent treatment decisions [26]. However, ctDNA lacks specificity, as elevated levels may also occur in inflammatory or benign conditions, and its clinical performance improves mainly when combined with other biomarkers [27].

Circulating RNAs, including microRNAs (miRNAs) and long non-coding RNAs (lncRNAs), also show potential as minimally invasive markers [28]. Several miRNAs, such as miR-21 [29], miR-29a [30], miR-92a [31], and miR-223 [32,33], are consistently overexpressed in CRC patients, while others (e.g., miR-145 [34], miR-18a [35]) are downregulated. Similarly, CRC-associated lncRNAs such as *CCAT1* [36] and *CCAT2* [37] demonstrate diagnostic and prognostic value, whereas others (e.g., *HOTAIR*, *PVT1*, *H19*) are shared across multiple cancers [38]. Despite encouraging results, both miRNAs and lncRNAs suffer from limited reproducibility and lack of standardization across studies, restricting their current clinical utility [39].

### 2.3. Circulating Tumor Cells

Circulating tumor cells (CTCs) are malignant cells shed from primary tumors or metastatic sites into the bloodstream. They have been identified across virtually all cancer types. In CRC, elevated CTC counts are generally associated with poorer prognosis and increased metastatic potential, highlighting their potential as prognostic biomarkers. Nevertheless, the diagnostic and prognostic value of CTCs remains a subject of debate, as studies to date have produced inconsistent or contradictory findings. Furthermore, false-positive results may arise due to the presence of benign cells that mimic CTCs, complicating their clinical interpretation [40].

### 2.4. Protein Biomarkers

Several serum proteins have emerged as potential biomarkers for CRC, most studied for diagnostic purposes and a few for prognostic use, aiding in predicting tumor progression and guiding treatment (Table 1). Currently, carcinoembryonic antigen (CEA) and carbohydrate antigen 19-9 (CA19-9) are the main protein biomarkers used clinically. However, both lack the specificity and sensitivity required for definitive CRC diagnosis, as elevated levels may also occur in conditions such as inflammatory bowel disease or pancreatic cancer. CA19-9, primarily a pancreatic cancer marker, exhibits low sensitivity for CRC (26–48%), whereas CEA is more useful for post-treatment monitoring and detecting disease recurrence [41].

To overcome these limitations, other members of the CA family, including CA72-4 and CA242, have been investigated. While their combined detection can improve diagnostic accuracy and aid prognosis [42], CA72-4 shows only 50% sensitivity due to elevations in other cancers and benign conditions, limiting its utility for CRC screening [43]. CA242 is typically used alongside CEA to enhance sensitivity and serve as a prognostic indicator [44].

In search of more robust alternatives, several promising protein candidates are under evaluation. For instance, tissue inhibitor of metalloproteinases-1 (TIMP-1) [45] and matrix metalloproteinases (MMPs) [46] have shown encouraging diagnostic accuracy in population studies, reflecting tumor invasion and tissue remodelling processes [47]. Notably, Gimeno-Garcia et al. evaluated MMP-9 as a diagnostic biomarker in a cohort of 25 CRC patients, 50 individuals with adenomas, and 75 healthy controls that were previously referred for colonoscopy. Using a cut-off value of 204 ng mL^−1^, MMP-9 achieved 67% specificity, 80% sensitivity, and an area under the curve (AUC) of 0.8, demonstrating considerable diagnostic potential. Likewise, members of the serpin family (e.g., SERPINA1, SERPINA3, and SERPINC1), exhibit altered expression patterns in CRC, highlighting their involvement in disease progression. For instance, elevated SERPINA1 levels are associated with poorer patient outcomes and promote CRC cell proliferation and migration. Moreover, the use of SERPINA1 has been statistically studied in a cohort of 15 CRC, 15 adenomas, 15 healthy controls with a validation test of 19 CRC patients and 21 healthy controls, obtaining 95% of sensitivity and specificity, an AUC of 0.97 with a cu-off value un serum of 817 µg mL^−1^. Additionally, some serpins, such as SERPINB5, may interact with established CRC biomarkers like CEA and are linked to more advanced disease stages [48].

To enhance the specificity of colorectal cancer (CRC) detection, systemic inflammatory markers have been extensively studied, observing a correlation between systemic inflammatory indicators and CRC susceptibility. Key indicators include C-reactive protein (CRP), interleukin-6 (IL-6), and tumor necrosis factor-alpha (TNF-α). CRP, an acute-phase protein, shows elevated serum levels in CRC patients; in a study of 164 CRC patients, 34 with adenomas, and 119 healthy controls, CRP achieved an AUC of 0.64, with 17% sensitivity and 90% specificity, indicating limited diagnostic utility alone [49]. IL-6, a pro-inflammatory cytokine, demonstrated improved diagnostic performance with an AUC of 0.794, 61% sensitivity, and 84% specificity [50]. TNF-α, a key signalling molecule, serves primarily as a prognostic marker: elevated levels correlate with advanced disease and poorer outcomes. In a study including 35 CRC patients, 20 with benign colorectal lesions, and 51 healthy controls, TNF-α reached an AUC of 0.9, with 80% sensitivity and 95% specificity [51].

To improve diagnostic performance, multi-analyte panels combining conventional and novel markers (e.g., CEA, CA19-9, CA24-2, and MIC-1, or CEA plus STK4) [52,53,54] have yielded higher accuracy in pilot studies, though validation in larger screening cohorts is still required [52,53,54]. Finally, HER-2/neu overexpression, detected in 2–5% of metastatic CRC cases [55], has emerged as a potential predictive marker, as it correlates with resistance to anti-EGFR therapies [56].

**Table 1:** List of serum proteins biomarkers for CRC diagnosis and their clinical parameters in terms of specificity, sensitivity, AUC and cut-off value.

Name	Clinically Relevant Parameters				Ref.
	Specificity	Sensitivity	AUC	Cut-Off Value	
CEA	89.2%	64.5%	0.789	3.36 ng mL^−1^	[57]
CA19-9	90.1%	47.8%	0.690	23.9 U mL^−1^	[57]
CA72-4	86%	50%	0.73	-	[43]
CA242	59.7%	66%	0.651	20 U mL^−1^	[58]
TIMP-1	95%	42–65%	0.83	-	[45]
CRP	90%	17%	0.64	14.6 ng mL^−1^	[49]
IL6	61%	84%	0.794	4.2 pg mL^−1^	[50]
TNF-α	85%	80%	0.90	-	[51]
STK4	92.3%	100%	-	-	[59]
SerpinA1	95%	95%	0.97	817 µg mL^−1^	[48]
MMP-9	67%	80%	0.8	204 ng mL^−1^	[60]

Taken together, these findings show that while a limited number of serum proteins (CEA, CA19-9) are currently validated for clinical use, several others (e.g., TIMP-1, MMP-9, SERPINA1) exhibit strong diagnostic potential and molecular characteristics compatible with aptamer recognition. Therefore, continued exploration of both established and emerging biomarkers is essential to expand the repertoire of aptamer targets suitable for minimally processed clinical samples.

## 3. SELEX for CRC Biomarkers

**Figure 2:**
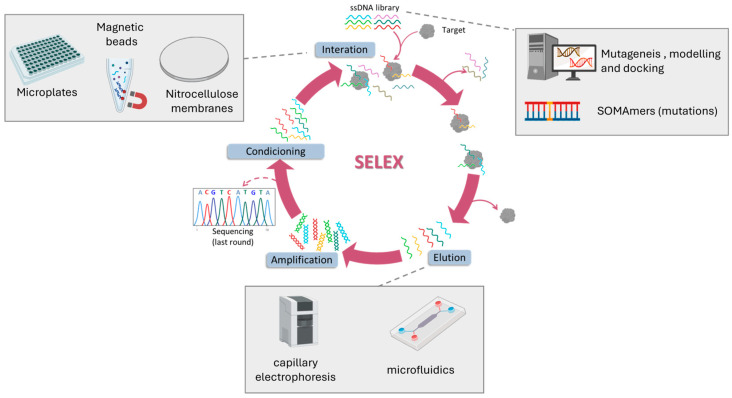
General scheme of the SELEX method and the different strategies employed to obtained aptamers against CRC biomarkers.

Aptamers have become invaluable tools for targeting disease-specific proteins, offering high precision in biomarker detection. The *Systematic Evolution of Ligands by EXponential enrichment* (SELEX) process enables the selection of high affinity aptamers through iterative binding and amplification cycles. Over time, SELEX methodologies have evolved from traditional separation techniques to modern, integrated platforms that combine automation, nanotechnology, and computational tools. In this section, we summarize recent advances in aptamer selection and optimization strategies, focusing on their progressive refinement toward CRC-relevant protein biomarkers (Figure 2).

Early SELEX methods relied on simple physical separation systems, such as nitrocellulose membranes (m-SELEX) or microplate immobilization, to separate bound from unbound sequences. Using these classical techniques, aptamers have been isolated against canonical CRC markers including CEA, CA72-4, and CA19-9, typically nanomolar affinities after multiple rounds of selection [61,62,63,64]. Despite their early successes, conventional SELEX was limited by labor-intensive workflows, lengthy selection cycles, and poor translation to physiological conditions. RNA aptamers, although exhibiting promising affinities, suffer from instability in biological fluids, and immobilization on microplate followed by proteolysis can distort target epitopes [63]. Furthermore, specificity against homologous proteins and robustness in complex biological matrices remain largely untested. Collectively, these limitations underscore why classical SELEX-derived aptamers have not yet bridged the gap to clinical application in CRC diagnostics.

To overcome these challenges, SELEX has progressively incorporated magnetic materials and microfluidic tools to improve efficiency and reduce selection bias. Magnetic beads for target immobilization (MBs-SELEX) markedly improved efficiency by enabling small sample volumes, rapid magnetic separation, stringent washing with minimal target loss, and direct polymerase chain reaction (PCR) amplification on bead surfaces, streamlining the selection process [64]. Using this strategy, Wang et al. obtained DNA aptamers against TIMP-1 in seven rounds with a dissociation constant (K_D_) of 0.41 nM, demonstrating recognition in serum [65]. Similarly, Minagawa et al. selected sub-nanomolar affinity aptamers against CRP [66] and Mashayekhi et al. for TNF-α [67]. A further refinement, *Magnetic-Assisted Rapid Aptamer Selection* (MARAS), introduced an externally applied rotating magnetic field to apply dynamic selection pressure, enabling the isolation of aptamers with distinct affinities to the target. Using this approach, an aptamer against C-reactive protein with an affinity of 25 nM was obtained [68]. MBs-SELEX has also been integrated with microfluidic or capillary electrophoresis platforms to reduce selection rounds. For example, Nagano et al. achieved aptamer selection against TNF-α in only three rounds using microbead-assisted capillary electrophoresis (MACE) SELEX [69], while Huang et al. selected combined incubation, magnetic extraction, and PCR amplification in a fully automated microfluidic MB-SELEX system [70].

Although these variants significantly increased efficiency and recovery of high-affinity candidates, several limitations remain. First, immobilization of target proteins on magnetic surfaces may induce structural alterations that bias aptamer selection, potentially limiting recognition of the native biomarker in physiological fluids. Second, the high affinities reported in controlled laboratory settings are often not reproduced in complex clinical matrices, raising concerns about real-world applicability. Finally, most reported aptamers, including those against TIMP-1, CRP, and TNF-α, have been validated only in small cohorts, and there is still little evidence that MBs-SELEX consistently yields aptamers ready for translational use. Thus, while magnetic bead-based strategies accelerate the SELEX process, further optimization and rigorous validation are required to confirm their utility in CRC diagnostics.

Parallel to these methods, chemical modification of nucleotides has given rise to *Slow Off-rate Modified Aptamers* (SOMAmers), representing a major leap in aptamer engineering. By incorporating side chain into DNA nucleotides, SOMAmers achieve enhanced molecular recognition, higher binding affinity and slower dissociation rates. These modifications also enable targeted post-SELEX optimization, improving functional activity and metabolic stability. Using this strategy, Gupta et al. successfully developed SOMAmers against IL-6 with sub-nanomolar affinity and with a great stability in human serum for more than 48 h [71]. This is particularly relevant for CRC, as reliable biomarker detection in complex fluids is a prerequisite for clinical translation.

The latest frontier in aptamer development lies in computational refinement. In silico tools such as mutagenesis, secondary structure modelling, and molecular docking simulations are increasingly used to streamline SELEX outcomes and reduce experimental cycles. A notable example is the affinity improvement of a DNA aptamer targeting CEA by generating mutant sequences and predicting their 3D RNA structures using Mfold and RNA Composer. The RNA models were converted to 3D DNA structures with Write, and their interactions with CEA were evaluated via ZDOCK. This approach successfully enhanced aptamer affinity to the low nanomolar range [72]. Although still in early stages, these approaches promise to streamline aptamer design and reduce experimental cycles.

Overall, SELEX technologies have evolved from classical separation methods to highly integrated, automated, and computationally guided pipelines. These innovations are beginning to deliver high-affinity aptamers against clinically relevant CRC biomarkers, setting the stage for their application in diagnostic assays and biosensing platforms.

## 4. Aptasensors for CRC Diagnosis

Aptamer-based biosensors (aptasensors) have rapidly evolved into diverse analytical formats for CRC detection. Beyond demonstrating the binding potential of selected aptamers, these platforms translate molecular recognition into measurable analytical signals with the sensitivity and reproducibility required for clinical application. Reported designs range from traditional formats, such as sandwich and direct assays, to advanced architectures integrating nanomaterials or nucleic acid-driven amplification strategies.

A clear trend can be observed among reported CRC aptasensors, which predominantly employ a direct assay configuration where the aptamer is immobilized on gold or nanomaterial-modified electrode surfaces. This arrangement allows for rapid detection and straightforward fabrication, but it is also more prone to matrix interference in complex biological fluids such as serum. Consequently, many studies rely on highly diluted serum samples to minimize nonspecific adsorption, which in turn reduces analytical sensitivity and limits clinical applicability. Although sandwich-type formats improve selectivity through dual recognition, they are generally more complex, involving additional preparation steps and longer assay times. Hybrid and nanomaterial-integrated designs (e.g., those employing AuNPs, graphene, or carbon nanotubes) have emerged as intermediate solutions that balance sensitivity and operational simplicity.

Most reported systems target protein biomarkers detectable in blood, and while a number of studies include recovery tests in spiked serum, only a few have evaluated real patient samples, restricting their translational relevance. Furthermore, despite achieving impressive limits of detection, some platforms remain over-engineered, making them less suitable for reproducible and point-of-care implementation.

Overall, a comparison of these approaches reveals key design rules for CRC aptasensor: direct formats favor speed and miniaturization, sandwich assays enhance selectivity, and hybrid nanomaterial platforms offer tunable performance but require simplified, standardized fabrication for clinical translation. Notably, most reported CRC aptasensors rely on electrochemical transduction (77%), followed by optical (20%) and, to a much lesser extent, mass-based approaches (3%), reflecting the clear advantages of electrochemical sensing in terms of sensitivity, portability, and cost-effectiveness, as already emphasized in previous reviews [11,73].

In addition to these design considerations, methodological rigor varies substantially among primary studies. Many CRC aptasensor reports lack appropriate negative controls to assess nonspecific binding, or rely on limited replicates, hindering statistical validation of results. Furthermore, essential analytical parameters such as the limit of detection, dynamic range, and inter-assay reproducibility are not always reported in a standardized format, complicating comparison between studies. Greater adherence to reporting standards, including the systematic use of negative and blank controls, independent replicates, and transparent analytical validation, will be critical to ensure reproducibility and accelerate clinical translation of aptamer-based biosensors.

### 4.1. Traditional Approaches

**Figure 3:**
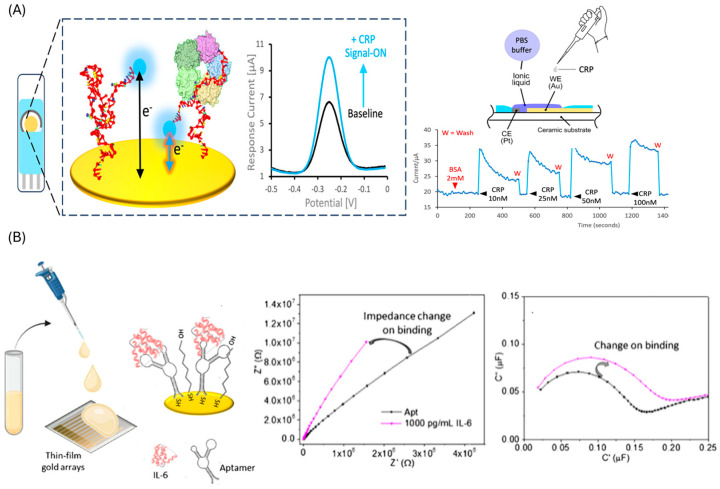
Traditional aptasensors. (**A**) Direct approach employing methylene blue labelled aptamer and electrochemical detection for CRP. Reprinted with permission from Ref. [81] (**B**) Direct approach employing aptamer modified gold electrodes and capacitive EIS for IL-6 detection. Reprinted with permission from Ref. [86].

Traditional aptasensor designs for CRC biomarkers (e.g., TIMP-1, IL-6, MMP-9, TNF-α, CRP, and CEA) rely on either sandwich or direct assay formats (Table 2 summarizes their analytical performance, including limit of detection, selectivity, detection time, and sample matrix; Figure 3).

Sandwich assays use an aptamer as the capture element and either a second aptamer or an antibody for detection, thereby improving selectivity through dual recognition. Representative examples include chemiluminescent detection of TIMP-1 [65], piezoelectric aptasensors for MMP-9 [74] and photoelectric strategies for split-type assays [75]. Although these designs can enhance analytical performance, they generally involve more complex fabrication steps, longer assay times, and, in hybrid formats, partially forfeit the advantages of fully aptamer-based systems.

Direct assays are more common due to their simpler construction and shorter analysis time. These rely on label-free techniques such as surface plasmon resonance (SPR) [76], or label-based techniques such as photoelectrochemical [77], fluorescence [78] and most predominantly electrochemical detection. In electrochemical formats, aptamers are typically immobilized on gold electrodes, and target binding is transduced as a change in signal. Strategies include methylene blue-labeled aptamers, which brings the reporter closer to the electrode upon target capture (demonstrated for IL-6 [79], TNF-α [80] and CRP [81,82]) (Figure 3A), or unmodified aptamers combined with solution-phase redox mediators, such as ferrocene, to monitor binding events (applied to CRP [83] and CEA [84,85]). Label-free capacitive approaches, including non-faradic impedance spectroscopy [86] (Figure 3B) and interdigitated electrode systems [87], have also been developed, particularly for IL-6 detection, with the advantage of eliminating the need for chemical labeling.

While sandwich and direct aptasensors have successfully demonstrated the feasibility of aptamer-based recognition for CRC biomarkers, key limitations persist. Sandwich assays achieve higher selectivity but at the cost of complexity, extended assay times, and, in some cases, antibody dependency. Direct assays offer speed and simplicity but are more vulnerable to nonspecific binding and matrix effects in clinical samples. Electrochemical approaches dominate the field owing to their sensitivity, portability, and compatibility with point-of-care miniaturization; however, challenges remain regarding long-term stability of the aptamer–electrode interface, reproducibility between fabrication batches, and robustness under physiological conditions (e.g., blood, variable temperatures). These issues highlight why, despite encouraging analytical performance in controlled studies, translation to clinical practice and large-scale validation remains limited.

To overcome these shortcomings, particularly in terms of sensitivity, stability, and real-world applicability, researchers are increasingly integrating nanomaterials into aptasensor designs. These materials enable signal amplification, improved robustness, and novel detection formats, representing the next major step in aptasensor development for CRC detection.

**Table 2:** Summary of aptasensors employing a traditional design.

Protein	Sensing Platform/ Assay Format	Detection Method	Linear Range/ LOD	Selectivity	Time	Samples	Stability	Ref.
CEA	ITO/Fe_3_O_4_@SiO_2_@CdS/Apt1/SiO_2_–Au-Apt2 Sandwich assay	Photo- electrochemical	1–6000 pg mL^−1^/ 0.3 pg mL^−1^	AFP, PSA, BSA, HCG	2 h	-	4 weeks	[75]
	PtμEs/Au/aptamer Direct assay	Electrochemistry (SWV)	10 pg mL^−1^–100 ng mL^−1^/ 7.7 pg mL^−1^	AFP, BSA, pancreatin, IgG	1 h	5 Blood samples	-	[84]
	IDE/Apt/MCH Direct assay	Electrochemistry (EIS)	2 pg mL^−1^–2 ng mL^−1^/ 2.4 pg mL^−1^	HSA, IL-6, SARS-CoV-2-S	20 min	1% spiked serum	-	[85]
	Apt + 1,1′-biphenyl]-4,4′-diyldiboronic acid Direct assay	Fluorescence	0.003–10 ng mL^−1^/ 1 pg mL^−1^	AFP, IgG, VEGF, BSA, CA125, IgE, PSA, PTK7	55 min	3 cancer serum	-	[78]
CRP	Au/Apt-MB/MCH Direct assay	Electrochemistry (SWV)	1–200 nM/ 20 nM	pfLDH, PCT	1 min	-	1 week	[81]
	Au/apt-MB/MCH Direct assay	Electrochemistry (SWV)	1 pM to 100 pM/ 1 pM	BSA, IgE	30 min	10% spiked serum	-	[82]
	Au/aptamer/MCH/BSA Direct assay	Electrochemistry (DPV)	0–1000 ng mL^−1^/ 1.84 ng mL^−1^	cTnT, cTnI	-	60 blood samples	-	[83]
	Fiber/Au/aptamer/MCH Direct assay	SPR	0–100 nM/ 1.7 nM	Hemoglobin, fibrinogen, cTn-I	-	-	-	[76]
	PTB7-Th/GCE-Au/Apt/HT Direct assay	Photo- electrochemical	1 pM–1000 nM/ 0.33 pM	BSA, IgG, Thrombin	30 min	-	-	[77]
IL-6	Au/apt-MB/MCH Direct assay	Electrochemistry (SWV)	50–200 nM/ 50 nM	TNF-α, HSA, H-calprotectin	-	-	7 days	[79]
	TFGA-Au/Apt/MCH Direct assay	Electrochemistry (non-faradaic EIS)	10–10,000 pg mL^−1^/ 10 pg mL^−1^	MMP3	30 min	Spiked serum	2 weeks	[86]
	IDE-strep/btn-apt Direct assay	Electrochemistry (current changes)	1 fM–100 pM/ 10 fM	-	5 min	1% spiked serum	-	[87]
MMP-9	Apt1/apt2-btn/strep Sandwich assay	Piezoelectric (QZM)	8.3–2075 ng mL^−1^/ 100 pg mL ^−1^	HSA, IgG	1 h	-	-	[74]
TIMP-1	MBs-strep/btn-apt/Ab-AE Sandwich assay	Chemi- luminescence	1–500 ng mL^−1^/ 1 ng mL^−1^	AFP, CEA, CA199, BSA, 8HIS peptide	-	40 serum samples	-	[65]
TNF-α	Au/apt-MB/MCH	Electrochemistry (SWV)	10–100 ng mL^−1^/ 10 ng ml^−1^	-	15 min	-	10 h (blood)	[80]

Ab: antibody; AE: acridinium ester; AFP: alpha feto protein; Apt: aptamer; Au: gold; BSA: bovine serum albumin; Btn: biotin; HCG: human chorionic gonadotropin; CA125; cancer antigen 125; CA199: cancer antigen 199, CEA: Carcinoembryonic antigen; CRP: C-reactive protein; cTnT: cardiac troponin; TcTn-I: troponin I; DPV: differential pulse voltammetry; EIS: electrochemical impedance spectroscopy; GCE: glassy carbon electrode; HCG: human chorionic gonadotropin; HSA: Human serum albumin; HT: hexanothiol; IDE: interdigitated electrodes; IgE: immunoglobulin E; IgG: immunoglobulin G; IL-6: interleukin 6; ITO: indium tin oxide; MB: methyleneblue; MBs: Magnetic beads; MCH: mercaptohexanol; MMP3: Matrix Metallopeptidase 3; PCT: procalcitonin; pfLDH: plasmodium lactate dehydrogenase; PTB7-Th: poly{4,8-bis[5-(2-ethylhexyl) thiophen-2-yl]benzo[1,2-b:4,5-b′]dithiophene-2,6-diyl-alt-3-fluoro-2-[(2-ethylhexyl)carbonyl] thieno[3,4-b]-thiophene-4,6-diyl}; PSA: prostate specific antigen; PTK7: Tyrosine-protein kinase-like 7; SPR: surface plasmon resonance; strep: streptavidin; SWV: square wave voltammetry; PtμEs: platinum microelectrode: TFGA: surface flexible thin film gold arrays chips; VEGF: vascular endothelial growth factor.

### 4.2. Nanomaterial-Based Sensors

Nanomaterials have emerged as a transformative component in the development of aptamer-based sensors, primarily due to their ability to improve sensitivity and robustness. Their high surface-to-volume ratio, tuneable optical and electronic properties, and versatile surface chemistry enhance aptamer immobilization, stabilize aptamer-target complexes, and enable efficient signal transduction. These features are particularly advantageous for detecting CRC biomarkers, where low analyte concentrations in complex biological samples demand exceptional analytical performance. In this section, we review the main classes of nanomaterials integrated into CRC biomarkers aptasensors, highlighting their specific roles in signal amplification and assay design.

#### 4.2.1. Gold Nanoparticles

**Figure 4:**
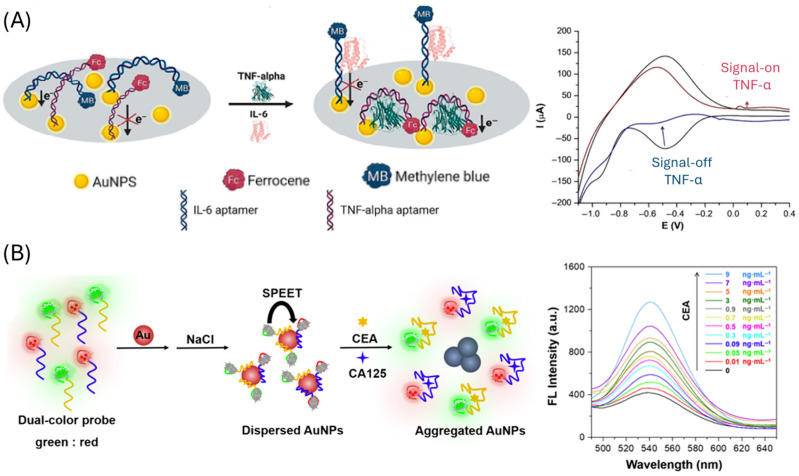
Gold nanoparticles-based CRC aptasensors. (**A**) approach employing AuNPs modified electrodes for the immobilization of the aptamer. Reprinted with permission from Ref. [89]. (**B**) Aptasensor based on salt-induced gold nanoparticles aggregation. Reprinted with permission from Ref. [99].

Gold nanoparticles (AuNPs) are among the most widely used nanomaterials in aptasensor design due to their high surface area and versatile binding molecules, which enable highly sensitive target detection [88]. Depending on the sensing strategy, AuNPs can serve either as the sensing phase or as signal labels (Table 3). As a sensing phase, AuNPs increase the available surface for aptamer immobilization (Figure 4A), thereby improving sensitivity in electrochemical [89,90,91,92,93], electrochemiluniniscence (ECL) [94], quartz crystal-microbalance (QCM) [95] and surface-enhanced Raman scattering (SERS) [96] platforms. When used as labels, AuNPs have been employed in multiple ways: conjugated to secondary aptamers and coupled with enzymes (e.g., horseradish peroxidase, HRP) for electrochemical detection of enzymatic products [97], as quenchers of quantum dot photoluminescence [98], and in aggregation-based assays (Figure 4B), where inhibition of nanoparticle clustering produces measurable fluorescence changes [99]. Additionally, AuNPs have been integrated with other nanomaterials like ZrO_2_ nanoparticles that improve the signal and stability of the sensor [100] and with Pt, mimicking the peroxidase activity [101].

Despite their versatility, AuNP-based sensors also face important challenges. Colloidal stability and aggregation under physiological conditions can compromise reproducibility, while variability in nanoparticle size, shape, and surface chemistry often introduces batch-to-batch inconsistencies that affect analytical performance. Moreover, although AuNPs enhance sensitivity, their incremental benefit over other nanomaterials is sometimes limited once baseline signal amplification is achieved. Most reported AuNP-aptasensors remain at the proof-of-concept stage, with scarce validation in real clinical samples, underscoring the need for further work to confirm their robustness in complex biological matrices.

**Table 3:** Summary of aptamer-based sensors employing gold nanoparticles.

Protein	Sensing Platform/ Assay Format	Detection Method	Linear Range/ LOD	Selectivity	Time	Samples	Stability	Ref.
CA125	DNA-AgNCs-apt+ AuNPs Direct assay	Fluorescence	0.01–2.0 U mL^−1^/ 0.015 U mL^−1^	DP, BSA, OVA, GOD, Tb	25 min	serum (*n* = 4)	2 h	[99]
CEA	Au/4-mercaptophenyl/AuNPs/apt Direct assay	QCM	0.1–25 ng mL^−1^/ 102 pg mL^−1^	AFP, CA-125, VEGF	50 min	Spiked serum	-	[95]
	GrSPE/AuNPs/apt/MCH Direct assay	Electrochemistry (EIS)	0.2–15.0 ng mL^−1^/ 0.085 ng mL^−1^	DHEA, AFP, Leptin, AA, BSA	1 h	-	-	[93]
	Au@AgNPs/4-MBA/apt Direct assay	SERS	0.01–200 ng mL^−1^/ 3.24 pg mL^−1^	AFP, DP, IgG, BSA	20 min	serum (*n* = 3)	-	[96]
	ITO/E-Si/ZrO_2_/AuNPS/apt Direct assay	Electrochemistry (DPV)	0.01 pg mL^−1^–100 ng mL^−1^ 42.504 fg mL^−1^	cTnI, CA199, NSE, l-cys	30 min	serum (*n* = 43)	-	[100]
	AuE/apt/MCH/CEA/apt2-Au@PtNPs Sandwich assay	Electrochemistry (amperometry)	0.1–100 ng mL^−1^/ 0.02–0.31 ng mL^−1^	AFP, AA, BSA, CA125,	2 h 30 min	spiked 20% serum	2 weeks	[101]
	DNA-AgNCs-apta+ AuNPs Direct assay	Fluorescence	0.01–0.9 ng mL^−1^/ 0.03–7.5 pg mL^−1^	DP, BSA, OVA, GOD, Tb	25 min	Serum (*n* = 4)	2 h	[99]
CRP	ePAD/EGaIn-PPD/AuNPs/Apt/ MCH/AuNPs Direct assay	Electrochemistry (DPV)	1–100 ng mL^−1^/ 0.039 ng mL^−1^	Cys, Glu, urea, Hcy	1 h	Saliva (*n* = 38)	-	[92]
	SPCE/AuNPs/Apt/casein/JNP-AuNPs/HRP Direct assay	Electrochemistry (amperometry)	10 pg mL^−1^–1 ng mL^−1^/ 3.1 pg mL^−1^	cTnT, cTnI, TB, HigG, HSA, Myo	1 h	serum (*n* = 3)	32 days	[97]
IL-6	Electrode/AuNPs/apt/MB Direct assay	Electrochemistry (CV)	5–5000 pg mL^−1^/ 1.6 pg mL^−1^	IL-1, IL-18, BSA, HSA	2 h	15 saliva, 15 sweat	3 days	[89]
TNF-α	Electrode/AuNPs/apt/ferrocene Direct assay	Electrochemistry (CV)	5–5000 pg mL^−1^/ 1.6 pg mL^−1^	IL-1, IL-18, BSA, HSA	2 h	15 saliva, 15 sweat	3 days	[89]
	GrSPECoHCF-AuNps/Apt/1-HT/Ab-HRP Direct assay	Electrochemistry (DPV)	1–100 pg mL^−1^/ 0.52 pg mL^−1^	APC, HSA, IgG	80 min	Serum (*n* = 3)	-	[90]
	GCE/AuNPS/Apt/MCH/TNF-α apt2-Ru(phen)2+/GO Sandwich assay	ECL	0.05–50 ng mL^−1^/ 36 pg mL^−1^	MMP-2, IL-2/6, BSA, IFN-γ	1h 45 min	-	-	[94]
	Fe_3_O_4_/AuNPs/Apt1/cApt-MB AuNPs Direct-displacement assay	Electrochemistry (SWV)	10 pg mL^−1^–100 ng mL^−1^/ 10 pg mL^−1^	IL-1, IL-2, IL-6, IL-12, IFN-γ	30 min	Spiked serum	-	[91]
	Quantum dot/apt/AuNPs Direct assay	FRET	0–22.3 nM/ 97.2 pM	HSA, CRP, transferrin, Tb	1 h	spiked serum (*n* = 5)	-	[98]

AA: ascorbic acid; AFP: alpha-feto protein; APC: activated protein-C; AgNPS: silver nanoparticles; Apt: aptamer; Au: gold electrode, AuNPs: gold nanoparticles; BSA: bovine serum albumin; CA: cancer antigen; cAPT: aptamer complementary; CEA: Carcinoembryonic antigen; CRP: C reactive protein; cTnI: troponin I; cTnT cardiac troponin; CV: cyclic voltammetry; Cys: cysteine; DHEA: dehydroepiandrosterone; DNA: deoxyribonucleic acid; DP: dopamine; DPV: differential pulse voltamety; EGaIn: eutectic gallium indium; EIS: electrochemical impedance spectroscopy; ePAD: electrochemical paper-based analytical devices; Glu: glucose; GO: graphene oxide; GOD: glucose oxidase; GrSPE: graphene screen printed electrodes; Hcy: homocysteine; HRP: horse radish peroxidase HSA: human serum albumin; HT: hexanothiol; IFN-γ: interferon-γ; IgG: immunoglobulin-g; IL: interleukin; ITO: indium tin oxide; JNP: janus particles; MB: methylene blue; MCH: mercaptohexanol; MMP: matrix metalloproteinases; Myo: myoglobin; NSE: neuron-specific enolase OVA: ovalbumin QCM: quartz microbalance; SERS: Surface-Enhanced Raman Scattering; SPCE: screen printed carbon electrodes; SWV: square wave voltammetry; TB: thrombin; VEGF: vascular endothelial growth factor; 4-MBA: 4-mercaptobenzoic acid.

#### 4.2.2. Carbon-Based Nanomaterials

Carbon-based nanomaterials have been extensively employed as sensing elements in biosensors owing to their large surface area, chemical stability, mechanical strength, biocompatibility, and facile surface functionalization [102]. They can be categorized by dimensionality into 0D, 1D, 2D, and 3D structures, a classification that helps highlight their different modes of action in aptasensor design. In the context of CRC, these nanomaterials have been applied both to canonical CRC biomarkers such as CEA and to inflammation-related proteins (e.g., CRP, IL6, TNF-α), which are increasingly recognized as contributors to CRC onset and progression.

Although several examples target non-CRC-specific inflammatory biomarkers, these studies demonstrate the broad adaptability of carbon nanomaterials for protein detection, and their underlying sensing principles are directly translatable to CRC biomarker assays (Table 4).

**Figure 5:**
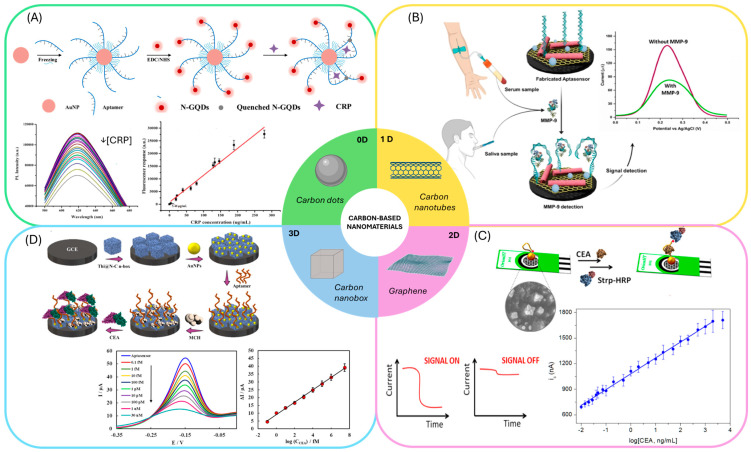
Aptasensors employing carbon-based nanomaterials: (**A**) Example of an aptasensors employing carbon dots and gold nanoparticles. Reprinted with permission from Ref. [104]. (**B**) Aptasensors employing carbon nanotubes for the immobilization of the aptamer. Reprinted with permission from Ref. [105]. (**C**) Sandwich aptasensors employing graphene modified electrodes. Reprinted with permission from Ref. [116]. (**D**) Aptasensor employing carbon nanoboxes to modify the electrode surface and gold nanoparticles for the immobilization of the aptamer. Reprinted with permission from Ref. [123].

0D materials such as carbon quantum dots (CQDs) doped with nitrogen and AuNPS were employed in Förster resonance energy transfer (FRET)-based aptasensors [103]. Similarly, graphene quantum dots combined with AuNPs have enabled the development of fluorescence aptasensors (Figure 5A) [104]. These optical strategies have been applied to markers associated with CRC-related inflammation, underscoring their potential for integration into CEA or CA19-9 detection formats.

1D materials, particularly carbon nanotubes (CNTs), both multi-walled and single-walled, are frequently used to modify electrodes in electrochemical aptasensors and as scaffolds in chemiluminescent sensors, enabling aptamer immobilization and direct target sensing. Multi-walled CNTs (MWCNTs) have been integrated with graphitic carbon nitride/zirconium dioxide (Figure 5B) [105], cobalt hexacyanoferrate [106], and flower-shaped Ag@ZIF-67 [107]. Single-walled CNTs (SWCNTs) have been used in combination with gold nanoparticles [108] and silver nanoparticle–mesoporous carbon composites [109]. Compared to quantum dots, CNTs are particularly effective in enhancing electrical conductivity and facilitating electron transfer, features that have been leveraged in electrochemical aptasensors for CRC-relevant biomarkers, including IL-6, TNF-α, and CEA.

Graphene and its derivatives, as 2D materials are the most widely exploited carbon-based nanomaterials for CRC biomarker detection. They are typically employed to modify electrode surfaces for aptamer immobilization [110,111,112,113]. Graphene can also form hybrid systems with Ag@Pt core–shell nanoparticles [114], AuNPs [115,116] (Figure 5C), silver nanoparticles [117], PdNPs [118], Cu_3_(PO_4_)_2_ Hybrid Nanoflowers [119], and even MWCNTs and AuNPs [120]. Additionally, graphene has been used as a carrier for generating electrochemical labels in combination with polymers [121]. This versatility stems from graphene’s exceptionally high surface area and ease of functionalization, which allow synergistic effects when integrated with metallic nanostructures, enhancing both conductivity and catalytic activity. Graphene-based systems have been among the most successful platforms for CEA detection, serving as proof-of-concept for CRC diagnostic biosensors.

Beyond planar systems, 3D carbon nanostructures introduce intrinsic cavities and hierarchical porosity that are advantageous for aptasensor applications. Carbon nanoboxes and carbon nanofibers exemplified this category [122]. Rahmati et al. exploited the hollow cavities of N-doped carbon nanoboxes to load thioine, the sensing molecule, while using AuNPs to immobilize the aptamer for CEA detection [123] (Figure 5D) Such designs enable the loading of both recognition elements and redox mediators within a single structure, facilitating high-performance sensing.

Carbon-based nanomaterials provide robust, versatile, and highly functional platforms for biosensor development. However, their intrinsic heterogeneity, potential cytotoxicity, and challenges in large-scale, reproducible synthesis remain limitations for clinical translation. In addition, while hybrid composites with metals or polymers enhance sensitivity, they also complicate fabrication protocols, potentially increasing variability between sensors. Future efforts should focus on standardizing these nanomaterial-based aptasensors for clinically validated CRC biomarkers, thereby accelerating their translation into diagnostic practice.

**Table 4:** Summary of the aptasensors for CRC biomarkers based on carbon nanomaterials.

Protein	Sensing Platform/ Assay Format	Detection Method	Linear Range/ LOD	Selectivity	Time	Samples	Stability	Ref.
CA19-9	GCE/GO-cMWCNTs/AuNPs cDNA-AQ/BSA/Apt-Fc Displacement assay	Electrochemistry (ACV)	1.0–1.0 × 10^6^ mUmL^−1^/ 0.65 mU mL^−1^	CA242, AFP, PSA	1 h	1% serum	15 days	[120]
CEA	MACNTs/apt/DNA-Ag@ZIF Displacement assay	Chemiluminescence	0.05–500 ng mL^−1^/ 4.53 pg mL^−1^	AFP, glu, adrenaline, Na^+^, BSA, Carbamide,	20 min	-	21 days	[107]
	GCE/AgNPs/SWCNT/MCF/Apt/BSA Direct assay	Electrochemistry (DPV)	1 fg mL^−1^–20 ng mL^−1^/ 0.30 fg mL^−1^	Glu, insulin, urea, gly, AA, HSA, Hb	1 h	Serum (*n* = 5)	16 days	[109]
	GO/AgNPS/aptamer Direct assay	Electrochemistry (DPV)	0.001–10 pg mL^−1^/ 0.5 fg mL-1	HER2, VEGF, IgG, MUC1 CFP10	-	Serum (*n* = 2)	-	[117]
	IDE/GO/apt1/BSA/CEA/apt2 Sandwich assay	Electrochemistry (LSW)	0.5–500 ng mL^−1/^ 0.96 ng mL^−1^	-	-	1% serum	-	[112]
	SPCE/rGO/AuNPs/casein/apt-btn/strep-HRP Direct assay	Electrochemistry (amperometry)	20 pg mL^−1^–2 μg mL^−1^/ 16 pg mL^−1^	cTnI, CRP, HSA, TBA, IgG	35 min	-	60 days	[116]
	PEDOT:PSS/GO/APTES/apt Direct assay	Electrochemistry (EIS)	0.77–14 ng mL^−1^/ 0.45 ngmL^−1^	BSA, PSA, insulin	-	Spiked serum	-	[113]
	Cu_3_(PO_4_)2HNF/GO/strep/btn-apt/Cu^2+^ Direct assay	Electrochemistry (SWV)	10 fg mL^−1^–500 ng mL^−1^/2.4 fg mL^−1^	PSA, TB, Hb	1 h	Serum	15 days	[119]
	GCE/Thi@N-C n-box/AuNPs /apt/MCH Direct assay	Electrochemistry (SWV)	0.1 fM–30 nM/ 0.03 fM	HCG, AFP, PSA	25 min	Spiked 0.1% serum	15 days	[123]
	GNSPE/PdNPs/apt/BSA Direct assay	Electrochemistry (DPV)	0.002–200 ng mL^−1^/ 1.0 pg mL^−1^	insulin, cys, glu, arg, gly, BSA, cholesterol	30 min	1% serum	5 weeks	[122]
	Au/apt/BSA/Apt2-CNTs- PFcGE Sandwich assay	Electrochemistry (SWV)	1 fg mL^−1^–10 ng mL^−1^/ 0.28 fg mL^−1^	DA, AA, UA, l-cys,	1 h	5% spiked serum	2 weeks	[121]
CRP	IDE/SWCNTs/Apt-AuNPs/ PEG-COOH Direct assay	Electrochemistry (SWV)	1 pM–10 nM/ 10 pM	Troponin I	30 min	-	-	[108]
	GCE/PDES/GO/AuNPs/Apt Direct assay	Electrochemistry (EIS)	0.001–50 ng mL^−1^/ 0.0003 ng mL^−1^	AFP, ly, UA	80 min	serum (*n* = 3)	10 days	[115]
	CSPE/CNFs-CHIT/RNA-apt/BSA/MB Direct assay	Electrochemistry (SWV)	1–150 pM/ 0.37 pM	HSA, IgG	1 h	Spiked 2.5% serum	15 days	[122]
	AuNPs/apt/N-GQDs Direct assay	FRET	0.2–300 ng mL^−1^/ 0.2 ng mL^−1^	BSA, cTNI, MYO	40 min	Serum	-	[104]
IL-6	GCE/MWCNTs/CoHCF/ /AuNPs/Apt/MCH Direct assay	Electrochemistry (DPV)	0.5–1000 pg mL^−1^/ 0.17 pg mL^−1^	BSA, IgG, CEA, MUC1	1 h	serum (*n* = 5)	15 days	[106]
	AuNPS-apt/NCD Direct assay	FRET	1.5–5.9 pg mL^−1^/ 0.84 pg mL^−1^	BSA, TNF-α	1 h	5% serum	-	[103]
MMP-9	GCE/MWCNTS-Gc_3_n_4_/OZrO_2_NPs/apt/BSA Direct assay	Electrochemistry (DPV)	50–1250 pg mL^−1^ 10.51 pg mL-1	leptin, TB, VEGF, MUC-1, IL-6, Apo-A1, NGAL	45 min	1% serum, 10% saliva	14 days	[105]
	IDE/Graphene/apt/6-FHH Direct assay	Electrochemistry (DPV)	10 fM–1000 nM/ 0.1 pM	-	-	Wound fluids	1 month	[110]
TNF-α	GO/strep/btn-PEG-Apt-Fc Direct assay	Electrochemistry (SWV)	5 –200 pg mL^−1^/ 5 pg mL^−1^	BSA, IL-6, IL-1β	1 h	Serum, sweat	30 days	[111]
	AuSPE/GO/Ag@PtNPs/Apt Direct assay	Electrochemistry (DPV)	0–60 pg mL^−1^/ 2.07 pg mL^−1^	BSA, Hb, cys, l-arg	2 h	serum (*n* = 3)	15 days	[124]
	SPCE/Au-GO/CS/apt/MCH/Apt-Ag@Pt-GRs Sandwich assay	Electrochemistry (DPV)	5–70 pg mL^−1^ 1.64 pg mL^−1^	BSA, Hb, L-cys, L-arg	2 h+ overnight	serum (*n* = 3)	14 days	[114]

AA: ascorbic acid; ACV: alternating current voltammetry; AFP: alpha-feto protein; AgNPS: silver nanoparticles; apt: aptamer; APTES: 3-Aminopropyl)triethoxysilane; Apo: apolipoprotein; AuNPs: gold nanoparticles; AuSPE: gold screen printed electrode; Arg: arginine; BSA: bovine serum albumin; btn: biotin; cDNA: complementary DNA; CEA: carcinoembryonic antigen; CNTs: carbon nanotubes; CRP: C-reactive protein; CSPE: carbon screen printed electrodes; cTnI: troponin I; Cys: cysteine; DPV: differential pulse voltammetry; EIS: electrochemical impedance spectroscopy; Fc: ferrocene; FH: ferrocenylhexanethiol; FRET: fluorescence resonance energy transfer; GCE: glassy carbon electrode; Glu: glucose; Gly; glycine; GO: graphene oxide; Hb: hemoglobulin; HCG: human chorionic gonadotropin HER-2: Human Epidermal growth factor Receptor 2; HNF: Hepatocyte nuclear factors; HRP; horse radish peroxidase; HSA: human serum albumin; IDE: interdigitated electrodes IgG: immunoglobulin G; IL: interleukin; LSW: linear sweep voltammetry; MACNTs: Magnetic Carbon nanotubes; MB: methylene blue; MCF: mesoporous carbon foam; MMP: matrix metalloprotease; MUC: mucin; MWCNTs: multi well carbon nanotubes; Myo: myoglobin; NCD: nitrogen dopped carbon dots; NGAL: lipocalin-2; N-GQDSNPs: nitrogen-graphene quantum dots nanoparticles; PEDOT:PSS: 3,4-ethylenedioxythiophene):poly(styrenesulfonate; PFcGE: poly(ferrocenyl glycidyl ether)-grafted; PEG: polyethylene glycol; PSA: prostate specific antigen; strep: streptavidin; SWCNTs: single well carbon nanotubes; SWV: square wave voltammetry; TNF-alpha: TB: thrombin; UA: uric acid; VEGF: Vascular Endothelial Growth Factor.

#### 4.2.3. Metal–Organic and Covalent Organic Frameworks

**Figure 6:**
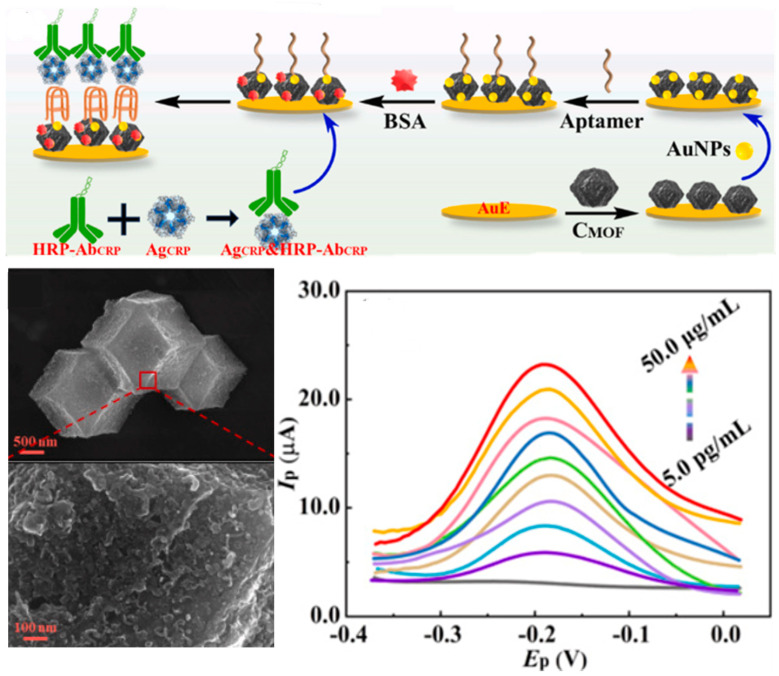
Sandwich aptasensors employing metal–organic frameworks (MOFs) in combination with gold nanoparticles for the electrochemical detection of CRP. Reprinted with permission from Ref. [127].

Metal–organic frameworks (MOFs) and covalent organic frameworks (COFs) are emerging as versatile porous nanomaterials for biosensing due to their high sorption capacity, tunable structures, and excellent electrocatalytic and electrochemical properties [125]. MOFs are commonly used to modify sensor substrates, enhancing surface area for aptamer immobilization and facilitating signal labelling. Different types of MOFs have been employed for developing aptasensors for CRC biomarkers (Table 5), such as ZIF-8@Au NPs@S QDs-MOFs enabling electrochemical, fluorescence and colorimetric detection [126]; nanoporous carbon-AuNP nanocomposites (Figure 6) [127]; Cu-MOF with intrinsic fluorescence emission and peroxidase-like activity [128]; zirconium–cobalt metal–MOFs [129], and zeolitic imidazolate MOFs [130]. Integration with other nanomaterials further enhances performance, such as Fe-MOFs combined with high-entropy alloy nanoparticles [131], AuNPs [132,133] and CdS quantum dots [134].

Similarly, covalent organic frameworks (COFs) have been employed as fluorescent quenchers [135] and integrated with 2D porphyrinic structures for photoelectrochemical transduction [136].

Both MOFs and COFs stand out as highly tunable platforms for aptasensor development, offering modular chemistry, high porosity, and multifunctional integration with other nanomaterials. However, despite their promising performance in proof-of-concept studies, challenges remain for their translation to robust CRC diagnostics. First, their structural and chemical stability in complex biological media is often limited, leading to partial degradation or loss of function. Second, fabrication protocols tend to be complex, with variability in crystallinity and particle size that may compromise reproducibility between sensor batches. Moreover, while their capacity for multimodal detection is attractive, this feature is not always aligned with clinical needs, where assay simplicity and robustness are usually prioritized. Taken together, MOFs and COFs provide a powerful toolkit for aptasensors, but their practical impact will likely depend on simplifying synthesis, improving biostability, and validating their performance in clinically relevant settings.

**Table 5:** Summary of aptasensors employing MOFs and COFs.

Protein	Sensing Platform/ Assay Format	Detection Method	Linear Range/ LOD	Selectivity	Time	Samples	Stability	Ref.
CEA	6FAM-apt/COF Displacement assay	Fluorescence	0.01–15 ng mL^−1^/ 5 pg mL^−1^	CA125, BSA, HSA, PSA, AGP, VEGF	55 min	serum	-	[135]
	GCE/HEANPs-Fe-MOF/apt/BSA Direct assay	Electrochemistry (DPV)	1 fg mL^−1^–10 μg mL^−1^ 0.115 fg mL^−1^	AFP, PSA, CP	35 min	10% plasma	12 days	[131]
	GE/rGO/AuNPS-MOF/apt Direct assay	Electrochemistry (EIS)	0.0025–0.25 ng L^−1^/ 0.8 pg L^−1^	MUC-1, HSA, IgG, HGB, BSA	1 h	Spiked serum	-	[132]
	AuE/ZrCo-MOF/apt/BSA Direct assay	Electrochemistry (DPV)	0.001–100 pg mL^−1^/ 0.35 fgmL^−1^	BSA, PSA, VEGF, IgG, MUC1, HER2, HSA, Tb, HGB	80 min	Spiked serum	15 days	[129]
	GCE/CdS QDs@MOF/TEOA@ AuNPs/apt/b-ME Direct assay	ECL	0.0001–10 ng mL^−1^ 0.085 pg mL^−1^	BSA, thrombin, lysozyme, trypsin	90 min	0.1% serum	-	[134]
	GCE/Fe-MOFAu@PDA/apt/BSA Direct assay	Electrochemistry (DPV)	1 fg mL^−1^–1 μg mL^−1^/ 0.33 fg mL^−1^	AFP, PSA	35 min	serum (*n* = 3; SA)	12 days	[133]
CRP	PCB/AuE/CMOF/AuNPs/aptamer/BSA/Ag-CRP+Ab-HRP Sandwich Assay	Electrochemistry (DPV)	5 pgmL^−1^–50 µgmL^−1^ 0.3 pg mL^−1^	IGF-I, GHBP, IL-6, IL-10, CA125, BSA	2 h	serum (*n* = 3)	21 days	[127]
	Cu-MOF/apt Displacement assay	Colorimetric/ Fluorometry	0.1–50 ng mL^−1^ 40 pg mL^−1^	Glu, GSH, AA, Fe, Cr, Ca, albumin,	15 min /8 h	10% serum	-	[128]
	ITO/NiS/pCOFs/AgNP/apt/MCH Direct assay	Photo-electrochemical	0.5–100 ng mL^−1^/ 0.1 ng mL^−1^	PSA, BSA, HCG, MC-LR	50 min	10% serum	20 days	[136]
MMP-9	GCE/rGO-AuNRs/cDNA/BSA/ apt-ZIF-8@Au NPs@S QDs Nanorod displacement	DPV Colorimetric Fluorescence	1 ng mL^−1^–10 μg mL^−1^ 10 fg mL^−1^–10 μg mL^−1^ 0.01–5 μM	annexin CAVIN2-, FEN1-, RAB26	40 min	-	30 days	[126]

AA: ascorbic acid; Ab: antibody; AFP: alpha-feto protein; apt: aptamer; AGP: Arabinogalactan protein; AuE: gold electrode; AuNPS: gold nanoparticles; AuNRs: gold nanorods; b-ME:β-mercaptoethanol; BSA: bovine serum albumin; CA: cancer antigen; CAVIN-2: caveolae-associated protein 2; cDNA: complementary DNA; CEA: carcinoembryonic antigen; COF: covalent organic framework; CP: calprotectin; CR; creatinine; CRP: c-reactive protein; DPV: differential pulse voltammetry; EIS: electrochemical impedance spectroscopy; FEN1: flap endonuclease 1; GCE: glassy carbon electrode; GE: graphene electrode; GHBP: growth hormone receptor; Glu: glucose; GSH: glutathione; HCG: human chorionic gonadotropin; HER2: Human Epidermal Growth Factor Receptor 2; HGB: hemoglobin; HRP: horse radish peroxidase; HSA: human serum albumin; IGF: Insulin-like growth factor; IgG: immunoglobulin G; ITO: indium tin oxide; MCH: mercaptohexanol; MC-LR: microcystin; MMP: matrix metaloprotease; MOF: metal–organic framework; MUC: mucin; NPs: nanoparticles; PCB: printed circuit board; PSA: prostate specific antigen; QDs: quantum dots; RAB26: ras-related protein; rGO: reduce graphene oxide; SA: standard addition; Tb: thrombin; VEGF: Vascular endothelial growth factor; 6-FAM: fluorescein.

#### 4.2.4. Other Nanomaterials

In the literature, other nanomaterials, including nanochannels, nanocomposites, nanocubes, nanosheets and quantum dots (QDs), have also been explored for the development of CRC biomarker aptasensors (Table 6).

**Figure 7:**
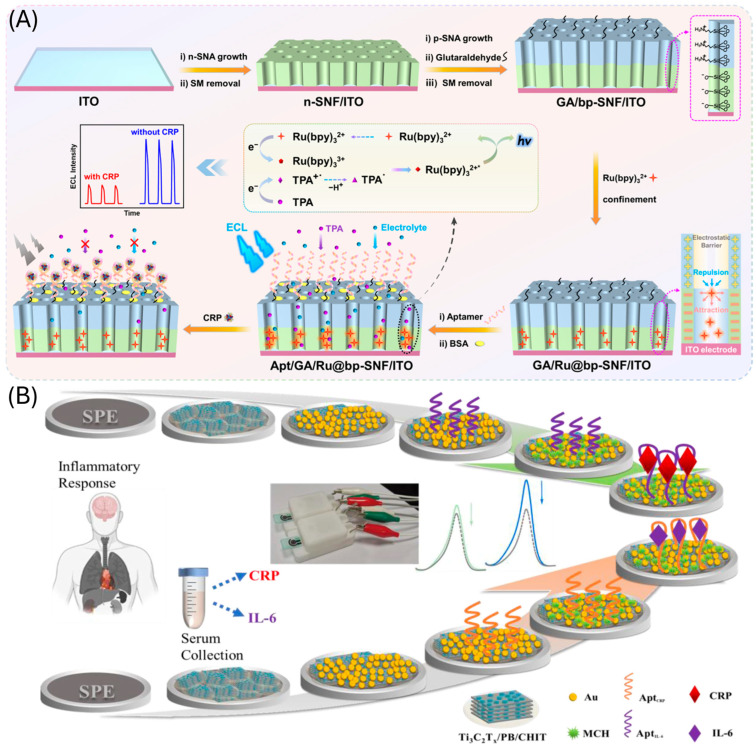
CRC aptasensors based on the use of (**A**) nanochannel for the immobilization of the aptamer and ECL detection. Reprinted with permission from Ref. [137] and (**B**) Ti3C2Tx and gold nanoparticles composites. Reprinted with permission from Ref. [141].

Vertically oriented ultrasmall nanochannels provide a high surface area, enhancing adsorption, reaction kinetics, and small-molecule transport. They also exhibit strong anti-interference and anti-fouling properties. These features make them highly suitable for ECL-based aptasensors, where the nanochannel architecture offers space to immobilize aptamers and localize ECL emitters near the electrode. In such systems, target binding to the aptamer typically leads to a decrease in the ECL signal [137,138,139,140] (Figure 7A).

Among nanocomposites, Ti_3_C_2_T_x_ has emerged as a dominant platform. It has been integrated with AuNPs [141] (Figure 7B) and MoS_2_ [142] to functionalize electrode surfaces, improve conductivity, and facilitate aptamer immobilization. Composites of Ti_3_C_2_T_x_ with silver/gold nanoparticles have also been used as electrochemical signal amplification strategies [143]. Another example is PdPt@PCN-224, a nanocomposite with excellent catalytic activity for the reduction of H_2_O_2_, enabling sensitive dual-signal output aptasensing [144].

Other nanostructures have also been investigated. CdS nanocubes have been applied in photoelectrochemical aptasensors as signal generators, combined with NiCo_2_O_4_-Au acting as a signal quencher [145]. Mn_3_(PO_4_)_2_/aptamer nanosheets have been used as labels in sandwich aptasensors [146]. Additionally, mercaptosuccinic acid capped nickel selenide quantum dots (MSA-NiSe_2_ QDs) have demonstrated promising electrochemical sensing capabilities [147].

While these other nanomaterials illustrate the breadth of strategies pursued in CRC aptasensor research, their application remains largely exploratory. Most studies report strong analytical sensitivity, yet reproducibility, biocompatibility, and long-term stability are seldom addressed. For instance, nanochannel-based systems offer elegant control over analyte transport but require highly controlled fabrication, limiting scalability. Similarly, advanced nanocomposites and QDs show signal amplification potential, but their complex synthesis routes and possible cytotoxicity may hinder translational progress. In this sense, these materials represent valuable platforms for proof-of-concept innovation, but their real impact will depend on balancing performance with cost-effectiveness, ease of fabrication, and clinical robustness.

**Table 6:** Summary of aptasensors employing different types of nanomaterials.

Protein	Sensing Platform/ Assay Format	Detection Method	Linear Range/ LOD	Selectivity	Time	Samples	Stability	Ref.
CEA	ITO/SNF-ngqdS/apt/BSA Direct Assay	ECL	0.001–10 ng mL^−1^/ 0.4 pg mL^−1^	Glucosa, NGAL, S100, AFP	-	2% FBS (SA)	-	[139]
	ITO/VMSF/PtNPs/Apt/BSA Direct assay	ECL	0 fg mL^−1^–100 ngmL^−1^/ 0.4 fg mL^−1^	AFP, CA19-9, S100, Hb, HSA, IgG, AA, UA, Glu, DA	90 min	2% spiked serum	-	[140]
	AuSPE/cDA-Fc/apt/PdPt@PCN-224 Displacement assay	Electrochemistry (DPV)	1 pg mL^−1^–100 ng mL^−1^/ 0.98 pg mL^−1^	Thrombin, PSA, HSA, AFP, IgG	30 min	Serum (*n* = 2)	-	[144]
CRP	ITO/bp-SNF/Ru(bpy)_3_^2+^/apt Direct assay	ECL	0.01–1000 ng mL^−1^/ 8.5 pg mL^−1^	CEA, SAA, CA-15-3, CA19-9, Glu, TRP	1 h	Spiked FBS	11 days	[137]
	ITO/SNF/AuNPs/Apt/BSA Direct assay	ECL	10 pgmL^−1^–1 μgmL^−1^ 4 pg mL^−1^	AFP, CA15-3, glu, l-serines, Na, K, Ca	30 min	2% spiked serum	15 days	[138]
	SPCE/Ti_3_C_2_T_x_/PBA/Chit/AuNP/Apt/MCH Direct assay	Electrochemistry (DPV)	0.1–15 μg mL^−1^/ 0.075 μg mL^−1^	Hb, cholesterol, cys, LPS, BSA, Glu	30 min	serum (*n* = 3)	30 days	[141]
	SPCE/MSA-NiSe_2_/QD/apt/BSA Direct assay	Electrochemistry (CC)	10–110 pg mL^−1^/ 2.80 pg mL^−1^	l-cys, BSA, cTnI,	-	10% serum (*n* = 3)	15 days	[147]
	SPCE/Ti_3_C_2_T_x_-AgNO_3_/AuNPS/apt/MCH Direct assay	Electrochemistry (DPV)	0.1–200 ng mL^−1^/ 41 pg mL^−1^	IL-6, BSA, Hb, Glu, Cys	40 min	Spiked serum (*n* = 2)	10 days	[143]
	GCE/ZIF67-C@AuNPs/apt/BSA/CRP/ Ab-HRP Sandwich assay	Electrochemistry (DPV)	10 pg mL^−1^–10 μg mL^−1^ 0.44 pg mL^−1^	IL-6/10, GHBP, IFN-γ, CA-125, BSA	1 h	Plasma (*n* = 4)	15 days	[130]
	GCE/PDA/AuNPs/Ab/BSA/CRP/aptamer@ Mn_3_(PO_4_)_2_ Sandiwch assay	Electrochemistry (DPV)	1 pg mL^−1^–1 ng mL^−1^ 0.37 pg mL^−1^	CEA, cTnI, NMP22, PSA	2 h 40 min	Serum (*n* = 8)	-	[146]
IL-6	SPCE/Ti_3_C_2_T_x_/PBA/Chit/AuNP/apt/MCH Direct assay	Electrochemistry (DPV)	0.01–100 ng mL^−1^/ 7 pg mL^−1^	Hb, cholesterol, cys, LPS, BSA, Glu	40 min	serum (*n* = 3)	30 days	[141]
	SPE/Ti_3_C_2_T_x_/MoS_2_/Au/NPs/apt/MCH Direct assay	Electrochemistry (CA)	5 pg mL^−1^–100 ng mL^−1^/ 2.9 pg mL^−1^	CRP, bovine l-cys, AA, BSA, UA	30 min	1% serum	-	[142]
TNF-α	ITO/CdS/Apt/MCH/TNF-α/NiCo_3_O_4_-Au-Apt2 Sandwich assay	Photoelectrochemistry	1 fg mL^−1^–1 ng mL^−1^/ 0.63 fg mL^−1^	h-FABP, CEA, l-cys	2 h 30 min	Spiked 10% serum	20 days	[145]

AA: ascorbic acid; Ab: antibody; AFP: alpha-feto protein; apt: aptamer; AuNPS: gold nanoparticles; AuSPE: gold screen printed, electrodes; BSA: bovine serum albumin; bp-SNF: bipolar silica nanochannel film; CA: cancer antigen; CEA: carcinoembryonic antigen; cDNA: complementary DNA: DA: dopamine; CHIT; Chitosan; CRP: C-reactive protein; cTnI: troponin I Cys: cysteine; DPV: differential pulse voltammetry; ECL: electrochemiluminescence; Fc: ferrocene; FBS: fetal bovine serum; GCE: glassy carbon electrode; GHBP; Growth Hormone Binding Protein; Glu: glucose; Hb: hemoglobin; hFBPA: heart-type fatty acid-binding protein; HSA: human serum albumin; HRP: horse radish peroxidase; IFN-γ: interferon-γ; IgG: immunoglobulin G; IL: interleukin; ITO: indium tin oxide; LPS: lipopolysaccharide; MCH: mercaptohexanol; MSA; mercaptosuccinic acid; NGAL: lipocalin-2; NGQDs: nitrogen-graphene quantum dots; NMP: Nuclear Matrix Protein NPs: nanoparticles; PBA: Phenylboronic acid; PDA: polymerized dopamine; PSA: prostate specific antigen; PtNPs: platinum nanoparticles; QDs: quantum dots; SA: standard additions; SAA: Serum Amyloid A; SPCE: screen printed carbon electrodes; S100: calcium related proteins; SNF: TNF-alpha: TRP: tryptophan; UA: uric acid; VMSF: vertically ordered mesoporous silica film.

### 4.3. DNA-Based Amplifications

To improve the sensitivity of aptamer-based sensors, DNA amplification strategies have emerged as effective alternatives. Since aptamers are typically single-stranded DNA (ssDNA), amplification methods such as the hybridization chain reaction (HCR) and exonuclease-assisted reactions can be directly integrated into aptasensors. In addition, DNAzymes provide another powerful means of enhancing sensitivity, as they can be readily incorporated into oligonucleotide-based signal amplification processes (Table 7).

**Figure 8:**
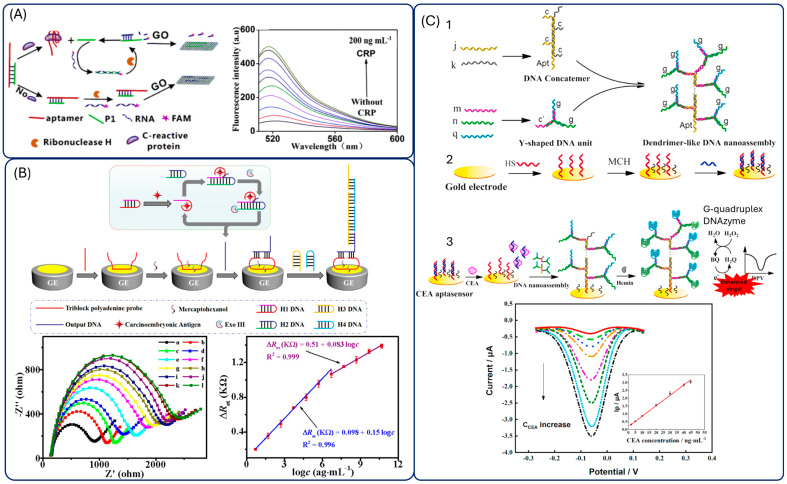
Aptasensors employing DNA-based amplification strategies. (**A**) Fluorescence sensor employing ribonuclease H reaction and graphene oxide. Reprinted with permission from Ref. [148]. (**B**) Electrochemical aptasensors employing exonuclease III reaction. Reprinted with permission from Ref. [157]. (**C**) Electrochemical sensor employing G-quadruplex formation and hemin catalysis. Reprinted with permission from Ref. [160].

One example is an RNase H-assisted DNA recycling strategy applied to a fluorescence assay for C-reactive protein (CRP). Binding of CRP to its aptamer releases the DNA strand P1, which hybridizes with a fluorescently labelled RNA. RNase H then selectively cleaves the RNA strand, generating fluorescent fragments that do not adsorb on graphene oxide, evading quenching and therefore producing fluorescence. This cycle repeats until enzyme depletion, resulting in strong signal amplification (Figure 8A) [148].

Endonuclease-assisted amplification has also been explored. A representative strategy employed magnetic beads functionalized with an aptamer and its complementary strand. Upon recognition of CEA the complementary DNA activated the endonuclease to cleave detection probes labelled with fluorophore–quencher pairs. Continuous cleavage resulted in the accumulation of fluorescent fragments in the supernatant, markedly enhancing the signal after magnetic separation [149].

Exonuclease-based amplification is among the most widely applied strategies, particularly Exonuclease III (Exo III) [150,151,152,153] and Exonucelase I [154,155,156]. For instance, Huang et al. combined Exo III-assisted target recycling with HCR amplification to achieve ultrasensitive electrochemical detection of CEA. The process involved multiple hairpin probes and enzymatic cleavage steps, producing abundant dsDNA polymers that interacted with a triblock polyadenine probe (TPP) immobilized on a gold electrode. The resulting steric hindrance and electrostatic repulsion effects enhanced the electrochemical impedance signal. This strategy achieves an impressive detection limit though at the cost of a long (>4 h) and complex experimental workflow (Figure 8B) [157].

Beyond enzymatic recycling, the structural features of aptamers have been exploited through DNAenzymes. Hemin intercalates into G-quadruplex (G4) motifs, forming a G4/hemin DNAzyme with horseradish peroxidase (HRP)-like activity [158]. This approach has been applied to CEA detection via photoelectrochemical [159] and fluorescence methods. However, the experimental protocol is excessively long (>8 h), and analyzing real samples requires a high dilution (1:1000), which complicates its use in hospitals and limits practical sample analysis. More sophisticated systems combine DNAzyme amplification with structural DNA nanodesigns: for example, a dendrimer-like DNA nanoassembly carrying multiple G-quadruplex motifs and a dangling aptamer. In the presence of CEA, the aptamer dissociates from the electrode, exposing the complementary DNA (cpDNA) to capture the nanoassembly. This arrangement harnesses the high density of G-quadruplex DNAzyme motifs to catalyze BQ efficiently, resulting in a significantly amplified electrochemical signal and a remarkably low detection limit of 0.24 ng·mL^−1^. The system demonstrates a synergistic strategy that combines target-triggered aptamer release with DNAzyme-based signal amplification for highly sensitive CEA detection (Figure 8C) [160].

Recent approaches increasingly combine multiple amplification mechanisms to maximize sensitivity. Examples include integrating rolling circle amplification (RCA) with exonuclease-assisted target cycling and G4/hemin catalysis [161] or coupling Exonuclease I-assisted recycling with ECL readout for CEA detection [162]. Hybrid systems combining Fe_3_O_4_@Au nanocomposites with Exo III and G-quadruplex/hemin amplification further highlight the trend toward synergistic amplification cascades [163].

Although DNA-based amplification strategies have achieved ultrasensitive detection limits, their complexity poses barriers to translation. Long incubation times, reliance on multiple enzymes and oligonucleotide constructs, and susceptibility to experimental variability limit their reproducibility and compatibility with point-of-care (POC) diagnostics. In particular, protocols often require hours of incubation and specialized expertise, which contrasts with the rapid and robust workflows needed in clinical settings. These strategies therefore remain highly innovative proof-of-concept approaches that demonstrate the versatility of aptamer chemistry but still face significant hurdles before practical implementation.

**Table 7:** Summary of aptasensors employing DNA-based amplifications.

Protein	Sensing Platform	Detection Method	Linear Range/ LOD	Selectivity	Time	Samples	Stability	Ref.
CEA	AuE/E1/MCH/T1-L2-L1/CEA ExoIII	Electrochemistry (DPV)	10 fg mL^−1^–50 ng mL^−1^/ 4.88 fg mL^−1^	TB, Hb, BSA	2 h 40 min	Serum (*n* = 3)	5 days	[153]
	AuE/CTF-ssDNA/MCH/rHp/ Cu_x_Mn_3_-x(HITP)_2_+ Exo-III	ECL	1 pg mL^−1^–50 ng mL^−1^/ 2.91 fg mL^−1^	AFP, CD63, NCL, CA-50	1 h 40 min	Serum (SA)	15 days	[150]
	AuE/AuNPs/Fc-HP2/MCH/exoIII/T4-DNA ligase+ Phi29-DNA polymerase+ dNTPs/MB	Ratiometric	1 pg mL^−1^–100 ng mL^−1^/ 0.59 pg mL^−1^	AFP, PSA, HSA	4 h 30 min	10% serum (*n*= 5)	-	[151]
	GCE/Au/cDNA/MCH/apt/ CEA+ExoI+ Ag^+^	ECL	100 ag mL^−1^–10 ng mL^1^/ 38.86 ag mL^−1^	HSA, PSA, AFP, IgG	1 h 30 min	1% serum (*n* = 3)	-	[154]
	AuE/AuNPs/hpDNA/MCH/Fc-apt/exoI+CEA	Ratiometric	10 pg mL^−1^–100 ng mL^−1^/ 1.9 pg mL^−1^	AFP, PSA, HSA	2 h	10% serum (SA, *n* = 3)	1 week	[155]
	AuE/AuNP/cpDNA-MCH/fcDNA ExoI+ CEA/tDNA/H1+H2+MB	Ratiometric	1 pg mL^−1^–100 ng mL^−1^/ 0.479 pg mL^−1^	AFP, PSA, HSA	1 h 30 min	10% serum (*n* =4)	21 days	[156]
	AuE/H2/apt/Exo-III+CEA/S1/S2/MB	Electrochemistry (DPV)	10 pg mL^−1^–100 ng mL^−1^/ 0.84 pg.mL^−1^	AFP, CA125, CA199,CA153	1 h 15 min	10% serum (*n* = 3)	7 days	[152]
	Photoanode: ITO/BVO/ZIS Photocathode: AuNP/CuBi_2_O_4_/ ITO/Apt1/CEA/Apt2/G4/hemin	Photo-electrochemistry	0.1 pg mL^−1^–10 ng mL^−1^/ 0.021 pg mL^−1^	AFP, BSA, IgG, PSA	2 h 20 min	Serum (*n* = 4)	600 s	[159]
	MCH/cpDNA/Au/Apt/MCH /cpDNA/Au	Electrochemistry (DPV)	2–45 ng mL^−1^/ 0.24 ng mL^−1^	BSA, HSA, IgG, CRP, CA125, AFP	3 h 24 min	Serum (*n* = 9)	31 days	[160]
	Aptamer/Harpins/DNA/hemin/G4+ HCR	Fluorescence	0.25–1.5 nM/ 0.2 nM	IgG, AFP, PSA	8 h 20 min	0.1% serum	-	[164]
	ExoI+ExoIII on MBs/AuE	Electrochemistry (DPV)	10 fg mL^−1^–100 ng mL^−1^/1.26 fg mL^−1^	BSA, AFP, UA CA125, Hb, AA	3 h	Spiked serum	30 days	[161]
	GCE/rGO-IL/PtNPs/Apt-G4/ hemin + exoI	Ratiometric- ECL	10 fg mL^−1^–10 ng mL^−1^/0.85 fg mL^−1^	HSA, AFP, PSA, IgG,	30 min	1% serum	10 days	[162]
	Fe_3_O_4_@AuNPs-S1-S2-S3 + exo III+ G4.	Electrochemistry (DPV)	0.1–200 ng mL^−1^/ 0.4 pg mL^−1^	HSA, Hb, l-cys, BSA	2 h 35 min	serum	-	[163]
	MNPs/strep/btn-cDNA/apt	Fluorescence	1–500 ng mL^−1^/ 0.7 ng mL^−1^	SCD146, IgG, BSA	3 h	plasma	-	[149]
	AuSPE/TPP+ exoIII	Electrochemistry (EIS)	Up to 1010 ag mL^−1^/ 0.39 ag mL^−1^	HSA, Hb, AFP, lysz ,BSAVEGF	4h 30 min	2% serum (SA)	20 days	[157]
CRP	GO+ aptamer+RNA-FAM+ ribonuclease H	Fluorescence	50 pg mL^−1^–100 ng mL^−1^/ 0.01 ng mL^−1^	d-dimer, MB, MUC1	50 min	2% serum, urine, saliva	-	[148]

AA: ascorbic acid; AFP: alpha-feto protein; AuE: gold electrode; AuNPs: gold nanoparticles; apt: aptamer; AuSPE: gold screen printed electrodes; BSA: bovine serum albumin; btn: biotin; BVO: BiVO_4_; CA: cancer antigen; cDNA: complementary DNA; CD63: Granulophysin; CEA: carcinoembryonic antigen; CRP: C-reactive protein; CTF: covalent triazine framework; dNTPS: nucleotides; DPV: differential pulse voltammetry; ECL: electrochemiluminescence; EIS: electrochemical impedance spectroscopy; Exo: exonuclease; FAM: fluorescein; Fc: ferrocene; Fc-HP: ferrocene-hairpin DNA; GCE: glassy carbon electrode; GO: graphene oxide; G4: g-quadruplex; Hb: hemoglobin; HCR: hybridization chain reaction; HITP: 2,3,6,7,10,11-hexaiminotriphenylene; HSA: human serum albumin; IgG: immunoglobulin G; ITO: indium tin oxide; l-cys: l-cysteines; Lyz: Lysozymes; MB: methylene blue; MBs: magnetic beads; MCH: mercaptohexanol; MNTPS: magnetic nanoparticles; MUC: mucin; NCL: nucleolin; PSA: prostate specific antigen; PtNPS: platinum nanoparticles; rGO/IL: reduced graphene oxide ionic liquid; rHp: residual hairpins strands; SCD146: soluble melanoma cell adhesion molecule; ssDNA: single stranded DNA; TB: thrombin; tDNA: trigger DNA; TTP: triblock polyadenine probe; UA: uric acid; VEGF: Vascular endothelial growth factor; ZIS: ZnIn_2_S_4_.

## 5. Adaptations to POC (Point-of-Care) Formats

Translating aptamer-based diagnostic tools into Point-of-Care (POC) applications requires balancing accuracy, speed, and usability. POC settings demand rapid results, streamlined workflows, and seamless integration into clinical environments, often operated by non-specialist staff. While highly sensitive, multistep amplification protocols can deliver outstanding sensitivity, they must demonstrate consistent reliability to be practical in real-world POC settings. Consequently, simplified assay formats, such as direct electrochemical or colorimetric platforms, are preferred as they reduce operational errors and enable reliable, real-time decision-making [165]. This section explores the strategies and innovations implemented to optimize sensing devices and protocols for effective and reliable POC deployment (Table 8).

**Figure 9:**
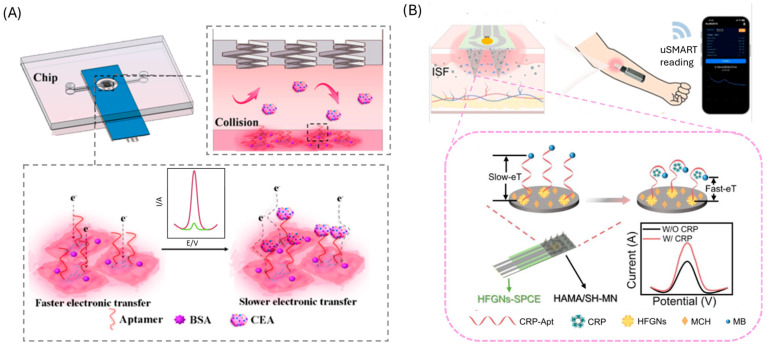
Adaptations of aptasensors for colorectal cancer diagnosis to a point-of-care format. (**A**) Aptasensor employing a microfluidic chip and electrochemical detection. Reprinted with permission from Ref. [167]. (**B**) Microneedle-based electrochemical aptasensors for the real time and continuous determination of CRP in interstitial fluid. Reprinted with permission from Ref. [172].

Microfluidic chips are gained traction as compact and automated systems that minimize reagent use while integrating sample pretreatment, separation, mixing, reactions, and detection steps [166]. These platforms enable fully integrated assays, combining sample handling, target enrichment, and sensitive detection on a single device. For example, Zhao et al. developed a microfluidic platform incorporating an electrochemical aptasensor for CEA. The device consists of a herringbone-embedded microfluidic chip above a sensor constructed from a MXene–carbon nanotube composite, providing strong signal amplification. The herringbone structures generate localized mixing, enhancing interactions between target molecules and the sensing interface. This design allows automated sample injection, efficient target capture, and sensitive electrochemical detection on a single, user-friendly platform [167] (Figure 9A). Similarly, Liu et al. integrated an electrode sensor array for TNF-alpha into a microfluidic device, enabling controlled fluid flow over multiple sensing elements [168]. Extending this principle to optical detection, Huang et al. coupled a SERS-based aptasensor with a microfluidic system integrating mixing chambers, serpentine channels, and magnetic separation. The system utilizes two nanomaterials with excellent SERS properties: gold-coated iron oxide particles (Fe_3_O_4_@AuNPs) and gold nanocages (AuNCs). In the assay, ssDNA1 and aptamers are immobilized on Fe_3_O_4_@AuNPs, while ssDNA2 and Raman tags are attached to AuNCs. Upon target binding, the aptamer detaches from Fe_3_O_4_@AuNPs, allowing ssDNA2 to hybridize with ssDNA1 and form the Fe_3_O_4_@AuNPs@AuNCs@SERS tag complex. This platform has been successfully applied to distinguish healthy individuals from cancer patients in just 15 min [169]. Microfluidic paper-based analytical devices (µPADs) represent another step toward affordable and disposable POC systems. The optical properties of gold nanoparticles (GNPs) functionalized with aptamers enable straightforward colorimetric readouts. Upon target binding, nanoparticle aggregation induces a visible color change, supporting rapid detection without the need for specialized equipment. For instance, Malekmohamadi et al. developed a µPAD aptasensor for CRP and IL-6, providing a low-cost approach to monitoring sepsis biomarkers [170].

Microneedles (MNs) have recently emerged as promising platforms for minimally invasive wearable and POC sensing technologies [171]. Using MNs, *Yuan* et al. developed an electrochemical aptamer-based sensor for real-time monitoring of C-reactive protein (CRP) in interstitial fluid (ISF) (Figure 9B). Integrated with a smartphone interface, the platform enabled real-time in vivo monitoring in animal models, demonstrating its potential for wearable healthcare technologies [172].

Collectively, these strategies highlight how aptasensor technology is moving closer to POC implementation by prioritizing automation, miniaturization, and user-friendliness. Microfluidic and paper-based systems reduce complexity while maintaining sensitivity, and wearable devices such as microneedles pave the way for continuous monitoring in outpatient or home settings. Nevertheless, widespread adoption still depends on demonstrating robustness in diverse clinical environments, scaling up fabrication, and validating performance across large patient cohorts.

**Table 8:** Summary of CRC aptasensors adapted to a point-of-care format.

Protein	Sensing Platform/POC Device	Detection Method	Linear Range/ LOD	Selectivity	Time	Samples	Stability	Ref.
CEA	Ti_3_C_2_-He@CCNT/Apt/BSA/CEA microfluidic chip	Electrochemistry (DPV)	10 pg mL^−1^–1 µg mL^−1^/ 2.88 pg mL^−1^	HSA, IgG, glu	1 h	Serum (*n* = 3)	-	[167]
CRP	uPAD/AuNPs/apt/salt paper-based + microfluidic	Colorimetric	0 -1000 mg L^−1^ 1.9 mg L^−1^/	BSA, IL-6, TNF-α	40 min	plasma (spiked)	1 year	[170]
	HFGN-SPCE/MB-apt Microneedles	Electrochemistry (SWV)	1 ngmL^−1^–100 μgmL^−1^/ 0.85 ng mL^−1^	UA, AA, Hb BSA, cTnI	seconds	In vivo (rats)	16 days	[172]
IL-6	Fe_3_O_4_AuNPs/apt/AuNC-RTG-cDNA Microfluidic chip	SERS	1 pg mL^−1^–1 µg mL^−1^ /0.178 pg mL^−1^	OPN, AFP	15 min	Serum (*n* = 30)	-	[169]
	uPAD/AuNPs/apt/salt paper-based + microfluidic	Colorimetric	1–25 ng L^−1^/ 0.07 ng L^−1^	BSA, CRP, TNF-α	40 min	plasma (spiked)	1 year	[170]
MMP9	Fe_3_O_4_AuNPs/apt/AuNC-RTG-cDNA Microfluidic chip	SERS	1 pg mL^−1^–1 µg mL^−1^/ 0.178 pg mL^−1^	OPN, AFP	15 min	Serum (*n* = 30)		[169]
TNF-α	Glass/Au/apt-MB/MCH + PDMS microfluidic device	Electrochemistry (SWV)	9–88ng mL^−1^/ 5.46 ng mL^−1^	IL-12, IL-6, IL-10, BSA, IFN-γ,	1 h	-	-	[168]

AA: ascorbic acid; AFP: alpha-feto protein; Apt: aptamer; Au: gold; AuNCs: gold nanocages; AuNPS: gold nanoparticles; BSA: bovine serum albumin; CCNT: carboxylic carbon nanotube; cDNA: complementary DNA; CEA: Carcinoembryonic antigen; CRP: C-reactive protein; cTnI: troponin I; DPV: differential pulse voltammetry; Glu: glucose; Hb: hemoglobin; He: hemin; HFGN: hierarchical flower-like gold nanostructure; HSA: human serum albumin; IFN-α: interferon alpha; IgG: Immunoglobulin G, IL: interleukin; MB: methylene blue; MCH: mercaptohexanol; MMP: matrix metalloprotease; OPN: Osteopontin; POC: point-of-care; µPADs: paper-based analytical devices; PDMS: Polydimethylsiloxane; RTG: Raman-tag; SPCE: screen printed carbon electrodes; SERS: Surface-Enhanced Raman Scattering; SWV: square wave voltammetry; UA: uric acid.

## 6. Market Transfer

For aptamer-based diagnostic tools to transition from research laboratories to clinical practice, several translational challenges must first be addressed. Stability in complex biological matrices, such as blood and stool, remains a major concern due to nuclease degradation and the presence of interfering biomolecules. This limitation can be mitigated by converting aptamers into peptide nucleic acids (PNAs) or by introducing chemical backbone modifications, which enhance enzymatic resistance while preserving binding specificity. Equally important is the need for validation using real patient samples and statistically robust cohorts, as many current studies rely on artificial matrices or spiked samples. Reproducibility across laboratories, standardisation of reporting practices, and evaluation under clinically relevant conditions are also essential prerequisites for regulatory acceptance.

Regulatory approval represents a critical milestone. In the U.S., the Food and Drug Administration (FDA) regulates in vitro diagnostic devices (IVDs) under the Center for Devices and Radiological Health (CDRH). Aptamer-based tests fall into this category and must demonstrate analytical validity (accuracy, precision, reproducibility), clinical validity (correlation with disease), and clinical utility (impact on patient outcomes) [173]. Depending on risk classification, regulatory clearance may proceed via the 510(k) pathway (when a predicate device exists) or via the more rigorous Premarket Approval (PMA) process [174].

In Europe, the In Vitro Diagnostic Regulation (IVDR) imposes comparable requirements, emphasizing clinical performance evaluation, risk stratification, and conformity assessment by notified bodies. For global commercialization, compliance with both FDA and IVDR standards, along with local regulatory frameworks, will be indispensable [175].

To date, only a few nucleic acid-based diagnostics (e.g., Cologuard, Guardant360) have successfully met these standards, providing a precedent for future aptamer-based tests. However, challenges remain, including the need for large-scale clinical validation, reproducibility across heterogeneous populations, and demonstration of cost-effectiveness.

Looking forward, the convergence of advanced sensor designs, nanomaterials, microfluidic integration, and wearable formats positions aptasensors as strong candidates for next-generation colorectal cancer diagnostics. Achieving market transfer will require not only technological refinement but also close collaboration between researchers, clinicians, regulatory bodies, and industry partners. If these hurdles are addressed, aptamer-based diagnostics have the potential to complement or even reshape existing CRC screening and monitoring strategies, bridging the gap between laboratory innovation and patient-centred healthcare.

## Data Availability

Not applicable.

## Abbreviations

The following abbreviations are used in this manuscript:

AA	Ascorbic acid
Ab	Antibody
ACV	Alternating Current Voltammetry
AE	Acridinium ester
AFP	Alpha-feto protein
AgNPS	Silver nanoparticles
AGP	Arabinogalactan protein
APC	Activated protein-C
Apo	Apolipoprotein
Apt	Aptamer
APTES	3-Aminopropyltriethoxysilane
Arg	Arginine
Au	Gold
AUC	Area under the curve
AuE	Gold electrode
AuNCs	Gold nanocages
AuNPs	Gold nanoparticles
AuNRs	Gold nanorods
AuSPE	Gold screen printed electrode
b-ME	β-mercaptoethanol
Bp-SNF	Bipolar silica nanochannel film
BSA	Bovine serum albumin
btn	Biotin
BVO	BiVO_4_
CA	Carbohydrate antigen
cAPT	Aptamer complementary strand
CAVIN	Caveolae-associated protein
CCNT	Carboxylic carbon nanotube
cDNA	Complementary DNA
CDRH	Center for Devices and Radiological Health
CD63	Granulophysin
CEA	Carcinoembryonic antigen
CHIT	Chitosan
CNTs	Carbon nanotubes
COFs	Covalent organic frameworks
CP	Calprotectin
cpDNA	Complementry DNA
CQDs	Carbon quantum dots
CR	Creatinine
CRC	Colorectal cancer
CRP	C-reactive protein
CSPE	Carbon screen printed electrode
CTCs	Ciculating tumor cells
ctDNA	Circulating tumor DNA
CTF	Covalent triazine framework
cTnI	Troponin I
cTnT	Cardiac troponin
CV	Cyclic voltammetry
cys	Cysteine
DA	Dopamine
DHEA	Dehydroepiandrosterone
DNA	Deoxyribonucleic acid
dNTPs	Nucleotides
DP	Dopamine
DPV	Differential pulse voltammetry
ECL	Electrochemiluminescence
EGaIn	Eutectic gallium indium
EIS	Electrochemical impedance spectroscopy
ePAD	Electrochemical paper-based analytical devices
Exo	Exonuclease
FBS	Fetal bovine serum
Fc	Ferrocene
Fc-HP	Ferrocene-hairpin DNA
FDA	Food and drug administration
FEN	Flap endonuclease
FH	Ferrocenylhexanethiol
FIT	Fecal immunotest
FRET	Förster resonance energy transfer
GCE	Glassy carbon electrode
GE	Graphene electrode
GHBP	Growth hormone receptor
Glu	Glucose
Gly	Glycine
GNPs	gold nanoparticles
GO	Graphene oxide
GOD	Glucose oxidase
GrSPE	Graphene screen printed electrodes
GSH	Glutathione
G4	G-quadruplex
Hb	Hemoglobulin
HCG	Human chorionic gonadotropin
HCR	Hybridization chain reaction
Hcy	Homocysteine
He	Hemin
HER-2	Human Epidermal growth factor Receptor 2
hFBPA	Heart-type fatty acid-binding protein
HFGN	Hierarchical flower-like gold nanostructure
HITP	2,3,6,7,10,11-hexaiminotriphenylene
HRP	Horse radish peroxidase
HSA	Human serum albumin
HT	Hexanothiol
IDE	Interdigitated electrodes
IFN	Interferon
Ig	Immunoglobulin
IL	Interleukin
ISF	Interstitial fluid
ITO	Indium tin oxide
IVDR	In vitro diagnostic regulation
IVDs	In vitro diagnostic devices
JNP	janus particles
K_D_	Dissociation constant
lncRNAs	Long non-coding RNA
LPS	Lipopolysaccharide
LSW	Linear sweep voltammetry
Lyz	Lysozymes
m-SELEX	Membrane-SELEX
MACE	Microbead-assisted capillary electrophoresis
MACNTs	Magnetic Carbon nanotubes
MARAS	Magnetic-Assisted Rapid Aptamer Selection
MB	Methylene blue
MBs	Magnetic beads
MCF	Mesoporous carbon foam
MCH	Mercaptohexanol
MC-LR	Microcystin
miRNAs	microRNAs
MMPs	Matrix metalloproteinases
MNs	Microneedles
MNTPS	Magneitc nanoparticles
MOFs	Metal–organic frameworks
MRD	Molecular residual disease
MSA	Mercaptosuccinic acid
MSI	Microsatellite instability
MUC	Mucin
MWCNTs	Multi-walled carbon nanotubes
Myo	Myoglobin
NCD	nitrogen dopped carbon dots
NCL	Nucleolin
NGAL	Lipocalin-2
N-GQDSNPs	Nitrogen-graphene quantum dots nanoparticles
NMP	Nuclear matrix protein
NPs	Nanoparticles
NSE	Neuron-specific enolase
OPN	Osteopotin
OVA	Ovalbumin
PBA	Phenylboronic acid
PCB	Printed circuit board
PCR	Polymerase chain reaction
PCT	Procalcitonin
PDA	Polymerized dopamine
PDMS	Polydimethylsiloxane
PEDOT:PSS	3,4-(ethylenedioxythiophene):poly(styrenesulfonate)
PEG	Polyethylene glycol
PFcGE	Poly(ferrocenyl glycidyl ether)-grafted
pfLDH	Plasmodium lactate dehydrogenase
PMA	Premarket Approval
PNAs	Peptide nucleic acids
PSA	Prostate specific antigen
PTB7-Th	poly{4,8-bis[5-(2-ethylhexyl) thiophen-2-yl]benzo[1,2-b:4,5-b′]dithiophene-2,6-diyl-alt-3-fluoro-2-[(2-ethylhexyl)carbonyl] thieno[3,4-b]-thiophene-4,6-diyl}
PTK7	Tyrosine-protein kinase-like 7
PtNPs	Platinum nanoparticles
QCM	Quartz crystal microbalance
QDs	Quantum dots
RAB26	Ras-related protein
RCA	Rolling circle amplification
rGO	Reduce graphene oxide
rGO/IL	Reduced graphene oxide ionic liquid
rHP	Residual hairpins strands
RTG	Rammna-tag
SA	Standard addition
SAA	Serum Amyloid A
SCD146	Soluble melanoma cell adhesion molecule
SELEX	Systematic Evolution of Ligands by Exponential Enrichment
SERS	Surface-Enhanced Raman Scattering
SOMAMERS	Slow Off-rate Modified Aptamers
SPCE	Screen printed carbon electrodes
SPR	Surface plasmon resonance
ssDNA	Single stranded DNA
strep	Streptavidin
SWCNTs	Single well carbon nanotubes
SWV	Square wave voltammetry
S100	Calcium related proteins
POC	Point-of-care
PtμEs:	Platinum microelectrode
TB	Thrombin
TcTn-I	Troponin I
tDNA	Trigger DNA
TFGA	Surface flexible thin film gold arrays chips
TIMP-1	Metalloproteinases-1
TNF-α	Tumor necrosis factor-alpha
TPP	Triblock polyadenine probe
TRP	Tryptophan
UA	Uric acid
VEGF	Vascular endothelial growth factor
VMSF	Vertically ordered mesoporous silica film
ZIS	ZnIn_2_S_4_
4-MBA	4-mercaptobenzoic acid
6-FAM	Fluorescein
µPADs	Microfluidic paper-based analytical devices
